# Advances in Data Pre-Processing Methods for Distributed Fiber Optic Strain Sensing

**DOI:** 10.3390/s24237454

**Published:** 2024-11-22

**Authors:** Bertram Richter, Lisa Ulbrich, Max Herbers, Steffen Marx

**Affiliations:** 1 Institute of Concrete Structures, TUD Dresden University of Technology, 01062 Dresden, Germany; bertram.richter@tu-dresden.de (B.R.);; 2 Hentschke Bau GmbH, Zeppelinstr. 15, 02625 Bautzen, Germany

**Keywords:** structural health monitoring, distributed fiber optic sensing, data quality, automation, data pre-processing, data filtering, software development, algorithm benchmarking

## Abstract

Because of their high spatial resolution over extended lengths, distributed fiber optic sensors (DFOS) enable us to monitor a wide range of structural effects and offer great potential for diverse structural health monitoring (SHM) applications. However, even under controlled conditions, the useful signal in distributed strain sensing (DSS) data can be concealed by different types of measurement principle-related disturbances: strain reading anomalies (SRAs), dropouts, and noise. These disturbances can render the extraction of information for SHM difficult or even impossible. Hence, cleaning the raw measurement data in a pre-processing stage is key for successful subsequent data evaluation and damage detection on engineering structures. To improve the capabilities of pre-processing procedures tailored to DSS data, characteristics and common remediation approaches for SRAs, dropouts, and noise are discussed. Four advanced pre-processing algorithms (geometric threshold method (GTM), outlier-specific correction procedure (OSCP), sliding modified *z*-score (SMZS), and the cluster filter) are presented. An artificial but realistic benchmark data set simulating different measurement scenarios is used to discuss the features of these algorithms. A flexible and modular pre-processing workflow is implemented and made available with the algorithms. Dedicated algorithms should be used to detect and remove SRAs. GTM, OSCP, and SMZS show promising results, and the sliding average is inappropriate for this purpose. The preservation of crack-induced strain peaks’ tips is imperative for reliable crack monitoring.

## 1. Introduction

The beneficial possibilities of distributed fiber optic sensors (DFOS) for the structural health monitoring (SHM) of infrastructure, such as bridges [1,2,3], tunnels [4,5,6], dams [7], pipelines [8], walls [9], or other engineering structures, are widely acknowledged [10,11,12]. It is anticipated that the maintenance costs are reduced by minimizing direct human interaction with the structure when DFOS are integrated into SHM systems with automated evaluation capabilities [13,14]. One of the benefits of coherent optical frequency domain reflectometry (c-OFDR)-based distributed strain sensing (DSS) is the ability to detect local damages [15,16,17,18]. However, apart from physical influences (which should be accounted for separately), raw DSS data are contaminated with different types of disturbances caused by the measurement principle, which complicate the evaluation by obscuring the sometimes weak signal caused by a structural changes (e.g., damages) [19,20,21]. With respect to real-world use cases, engineers will have to accept low-quality data even with the best possible setup available due to challenging measurement conditions, such as dirt, moisture, ambient vibrations [22], sensor degradation due to chemical or mechanical influences [23,24], transversal pressure, and further local effects [25]. A reliable evaluation of DSS data requires the reliable elimination of those disturbances caused by the measurement principle  [26], as well as compensation of the physical influences. This study focuses on removing measurement principle-related disturbances from the raw data. The data cleaning step is called pre-processing within this study as it is an upstream process of data evaluation for the structural information extraction workflow.

While evaluating data, it was observed that the pre-processing capabilities at hand, tailored towards DSS data, were insufficient. The process required manual intervention, an iterative procedure (trial and error), or eyeballing. As pre-processing is an integral part of the data evaluation workflow, the aforementioned benefits can only be achieved if pre-processing can be carried out reliably and automatically. To tackle the challenge of DSS data pre-processing, the following research questions are investigated. Each research question is examined in one of the remaining sections.

- *Which types of disturbances exist? What are their potential causes and characteristics? How are they dealt with by the DFOS community?* These questions are answered through a literature review in Section 2.
- *How can the pre-processing of DSS data be generalized?* In Section 3, pre-processing tasks are categorized, and a modular, flexible pre-processing workflow is presented.
- *Which advanced pre-processing algorithms exist?* In Section 4, selected pre-processing algorithms are presented, their respective advantages and disadvantages are discussed, and modifications are proposed.
- *How reliable are the selected pre-processing algorithms on DSS data acquired in crack monitoring scenarios?* In Section 5, the benchmark prerequisites are presented. The benchmark was carried out on artificial data, taking into account different scenarios. A simple functional model—based on the DFOS sensitivity—is introduced to simulate crack-induced strain peaks. Performance measures for the pre-processing are presented. In Section 6, the benchmark results are discussed.

Limitations are highlighted in Section 6.4. Finally, concluding remarks are presented in Section 7.

## 2. Measurement Disturbances in DFOS Data

**Figure 1:**
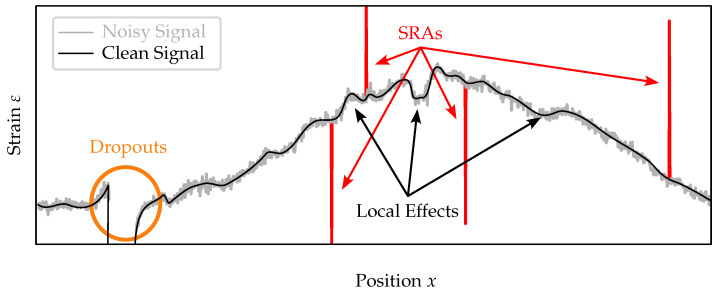
Schematic of the different measurement disturbances: SRAs, dropouts, and noise. The variation in the signal marked as local effects is not caused by the measurement principle.

This section provides an overview over three different disturbance types—strain reading anomalies (SRAs), dropouts, and noise—present in DSS measurements. A schematic of the disturbances is shown in Figure 1. For each disturbance type, causes and specific characteristics are described, followed by an overview of coping strategies actually employed by other researchers. Although the causes and characteristics of the disturbances are discussed specifically for the c-OFDR-based optical distributed sensor interrogator (ODiSI) manufactured by Luna Inc. (Roanoke, VA, USA), the coping strategies should be transferable to other DSS methods.

### 2.1. Strain Reading Anomalies (SRAs)

#### 2.1.1. Characteristics and Causes

SRAs are misreadings, characterized by outliers of implausible high or low values; see Figure 1. SRAs are caused by a failed cross-correlation between the reference and measurement signal in the ODiSI [27]. There are two types of SRAs. (i) Harmless strain reading anomalies (HL-SRAs) are isolated glitches in the strain readings in space and time. (ii) In contrast, harmful strain reading anomalies (HF-SRAs) persist (in time) after their first occurrence.

#### 2.1.2. Coping Strategies

It is recommended to convert SRAs to dropouts [19,27]. The conversion of a SRA to a dropout is a form of outlier exclusion, which is common practice in data science (replacing an “obviously wrong” value with an “unknown” value). This SRA elimination can be divided into three steps: (i) Possible SRA candidates are identified in the first step. Ideally, no false negatives (SRAs categorized as normal data) remain, at the cost of some false positives (normal data categorized as SRAs). (ii) False positives are eliminated in the second step—candidate verification. (iii) In the last step, identified SRAs are replaced with not a number (NaN). Afterwards, the resulting dropout can be dealt with as discussed in Section 2.2.

The ODiSI manufactured by Luna Inc. implements the conversion of SRAs to dropouts with spectral shift quality (SSQ) as a reliability/outlier measure [21,22,28]. SSQ evaluates the plausibility of the cross-correlation between the spectra of the reference reading and the current measurement [29,30,31,32]. If the SSQ falls below a certain threshold, the reading should be considered an outlier (SRA) and ignored in further processing steps (set to NaN). Different SSQ thresholds are recommended (depending on the use case), e.g., <0.2 [29] or <0.15 [31]. However, SSQ has been shown to be an insufficient measure in [27,31]. In earlier series of the ODiSI, SSQ could be exported along with the raw measurements and the threshold was configurable [28], which is no longer possible with the ODiSI 6100 series [22]. The SRAs contained in the exported data are exclusively false negative classifications obtained using the SSQ method.

Other SRA elimination approaches for DSS data employ outlier detection algorithms, such as (i) based on a local outlier factor [32], (ii) the geometric threshold method (GTM) [19], (iii) the polynomial interpolation comparison method [19], (iv) based on the deviation from sliding median filtering [33], (v) *z*-score-based algorithms [21], or (vi) the outlier-specific correction procedure (OSCP) proposed for the processing of surface scans [34,35].

### 2.2. Dropouts

#### 2.2.1. Characteristics and Causes

Dropouts are missing values represented by NaN in the exported DSS data; see Figure 1. A reading becomes a dropout if it is flagged as anomalous by the ODiSI’s built-in SRA detection method (SSQ) [21]. However, large total deviations from the reference state or large gradients cause false positives. According to the manufacturer’s manual [22], the limit for the absolute strain difference compared to the reference state is approximately 12,000 με. The permissible strain difference between two readings is specified in [28] as 517 με for a gage pitch of 1.25 mm and 129 με for a gage pitch of 5 mm. Exceeding these limits results in dropouts. The influence of dropouts on c-OFDR-based crack monitoring is discussed in [36].

#### 2.2.2. Coping Strategies

There are two strategies to cope with the aforementioned limitations: (i) avoid the technical conditions leading to dropouts and (ii) reconstruct missing values by interpolation during the pre-processing stage.

The a priori approach is to avoid exceeding the technical limits of the ODiSI in the first place. Here, the strain transfer between the host material and the optical fiber becomes crucial [37,38,39]. The DFOS type and installation method must always be selected according to the specific measurement task. In [36], an empirical method is proposed for the design of DFOS monitoring systems, accounting for the DFOS type, installation method, crack pattern, and measurement settings.

The a posteriori approach is to eliminate dropouts from the raw data during the pre-processing stage by (i) replacing them with estimated values using interpolation [20] or by (ii) excluding the dropout readings from data (which might be equivalent to interpolation depending on the later evaluation steps) [16].

Several approaches for interpolation can be applied. Linear interpolation between the remaining valid readings along one axis (either space or time) is the standard approach [15,16,19,20,29]. However, linear interpolation results in a drastic underestimation of the crack widths if the tip of the strain peaks is missing due to the technical limits of the ODiSI. Interpolation with higher-degree polynomials are discussed in [27]. However, polynomials can approximate complex strain profiles only locally due to their trend towards positive or negative infinity when the argument increases indefinitely. Piece-wise polynomial functions—so-called splines—can overcome this limitation of polynomials. One specific spline type—Akima splines—have the advantages to not require assumptions, are fast, and do not show overshooting behavior [40]. Hence, Akima splines are used to interpolate dropouts in [41].

DSS data sets are time-series of one-dimensional spatially distributed data. Due to the incompatible units (meter and s), there is no meaningful common metric of the space and time. Hence, two-dimensional interpolation methods (e.g., as proposed in [34] for two-dimensional spatial data using distance weighting) are not directly transferable to DSS data.

### 2.3. Noise

#### 2.3.1. Characteristics and Causes

Noise is a high-frequency, low-amplitude component usually present in measurement data [34,42]; see Figure 1. Noise should not be confused with local effects, such as the “concrete signature” caused by inhomogeneities and variations in Young’s modulus within the concrete matrix [43] or local strain variation due to bond interactions [25,33,44]. Noise fluctuates in both dimensions (spatial and temporal), in contrast to those local effects, which are relatively stable in time. The intensity and characteristics of noise depend on several factors, such as the quality of fiber connectors, splices, or fibers; measurement settings and the operational mode of the ODiSI, [22]; vibration [45]; or loss (e.g., caused by small bending radii) [46].

#### 2.3.2. Coping Strategies

Noise is commonly reduced by applying low-pass filters [47,48,49]. Examples for low-pass filters used for smoothing DSS data are the sliding average [25,33,50,51], the sliding median [52], locally weighted scatterplot smoothing (LOWESS) [53], Butterworth filters [20,54], Bessel filters [20], Savitzky-Golay filters [20,49], wavelet filtering [55], and extremal filtering [56]. However, filtering might be of questionable benefit or even detrimental (by distorting the signal), especially when applied excessively [33,47]. Another approach is to apply aggregate functions to combine several readings when reducing data size, also called “ensemble averaging” [20,39,47]; see Section 3. However, ensemble averaging reduces the effective measurement rate or spatial resolution [55,56].

## 3. A Generic Workflow for Pre-Processing of DFOS Data

An appropriate pre-processing strategy depends on the data at hand and the goal of the data evaluation. Hence, the individual pre-processing steps may change for each application. Therefore, a generic pre-processing workflow should fulfill the following requirements.

Firstly, it should be possible to freely select and configure algorithms for each step independently from each other. Secondly, the workflow should allow us to carry out the chosen algorithms in an arbitrary order. Thirdly, the workflow should respect that the data array’s shape (number of rows and columns) might be changed by any of the algorithms. Finally, each algorithm should be able to deal with both one-dimensional data (distributed in time or space) and two-dimensional data (distributed in time and space). The operation of inherently one-dimensional algorithms on two-dimensional data should be applied in a transparent way along one axis—row-wise (along space domain) or column-wise (along time domain).

**Figure 2:**
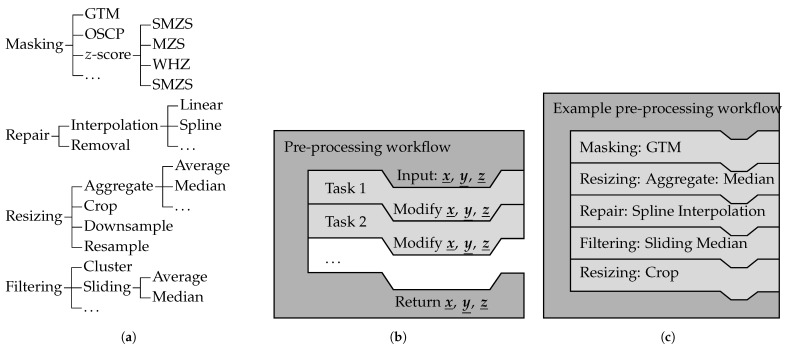
The task and workflow duality implemented by the pre-processing module of fosanalysis. (**a**) Class inheritance hierarchy of task objects. (**b**) Structure of a pre-processing workflow object. (**c**) Pre-processing workflow with an exemplary sequence of task objects.

To meet the previously stated requirements, the free open source software framework fosanalysis implements from its version v0.4 onwards (available at https://github.com/TUD-IMB/fosanalysis, published and accessed 18 November 2024) the concepts of task objects and workflow objects to achieve a flexible pre-processing pipeline. The This concept is visualized in Figure 2.

Task objects implement one specific algorithm, its methods, settings, and parameters. Task objects are interchangeable because they expose the same interface. The group hierarchy of the pre-processing tasks is shown in Figure 2a. The terminology for pre-processing tasks is as follows:

**Masking**  algorithms identify SRAs using anomaly detection. For each element, a decision is made of whether this element is considered anomalous (SRA) or a normal data point. The values of elements considered anomalous are replaced with NaN. The data array’s shape (number of rows and columns) is not changed. In this paper, three SRA detection algorithms are presented: (i) the GTM discussed in Section 4.1, (ii) the OSCP discussed in Section 4.2, and (iii) the sliding modified *z*-score (SMZS) discussed in Section 4.3.
**Repair**  algorithms replace dropouts with values estimated from the surrounding data, usually by interpolation. The data array’s shape (number of rows and columns) might be changed. Linear interpolation and spline interpolation for dropout reconstruction are addressed in Section 6.3.
**Filtering**  algorithms modify the values of the data to smooth the signal by reducing high-frequency components (low-pass filters). The data array’s shape (number of rows and columns) is not changed. In this paper, three filter algorithms are presented: (i) the sliding average, (ii) the sliding median, and (iii) the cluster filter; see Section 4.4.
**Resizing**  algorithms change the data array’s shape. They can be divided into three groups: (i) Downsampling is used to reduce the data volume by either taking only every *m*th element or consolidating several elements into a single element using aggregate functions (e.g., from two-dimensional to one-dimensional or even to a singleton) [47]. (ii) Resampling is used to obtain readings at different sampling points (in time and/or space) compared to the original sampling points by means of interpolation. (iii) Cropping is used to restrict the data to a specific area (i.e., cut away uninteresting parts).

Note that algorithms can have effects associated with multiple groups, e.g., downsampling will also reduce the noise or a filtering algorithm might cancel out SRAs too.

Workflow objects are containers that implement the pre-processing pipeline and coordinate multiple task objects; see Figure 2b. A workflow object holds the order of the tasks and calls them sequentially. This container will pass its input to the first task object and pipe the output of one task object to the input of the next task object. Because the tasks are interchangeable, any sequence of pre-processing tasks can be established. An example for such a workflow is shown in Figure 2c.

While the actual sequence of the pre-processing steps might depend on the data set and the use case, particular sequences make sense:

- SRA removal should be followed up by dropout elimination.
- SRA removal should be carried out before smoothing.
- Downsampling with the average should be preceded by SRA removal.
- Downsampling with the median might not need to be preceded by SRA removal since it is more stable against outliers and might stabilize the signal instead.
- Depending of the density of the target points, resampling might be preceded by a combination of the other pre-processing steps.

## 4. Pre-Processing Algorithms

This section presents three masking algorithms (for SRA removal): (i) GTM in Section 4.1, (ii) OSCP in Section 4.2, (iii) SMZS in Section 4.3. It also presents one filtering algorithm (for smoothing): the cluster filter in Section 4.4. Implementations for all four algorithms were made available with fosanalysis. Each of the following subsections is structured into three parts: (i) a presentation of the algorithm’s original concept, (ii) a discussion of the algorithm’s advantages and disadvantages, and (iii) a presentation of modifications made to the algorithm to overcome the disadvantages.

Note that zero-based numbering (as is common in the Python 3 programming language) is used for all algorithm descriptions. Assignment statements are denoted by a left pointing arrow (e.g., a←0 means “the variable *a* is set to 0”, and a←a+1 means “the value of *a* is incremented by one”), while equality comparison statements are denoted by the equals sign (e.g., a=b? denotes a check, whether the variables *a* and *b* hold the same value).

All presented algorithms operate on one-dimensional strain data. The operation of two-dimensional strain data (time-series of spatially distributed measurements) is supported by carrying out the one-dimensional operation for each row or column independently. The algorithms can be applied both spatially (i.e., along the DFOS’ length for one specific time stamp) or temporally (i.e., on a time-series of a specific position in the DFOS). However, the relation along the DFOS’ length is governed by mechanical laws, whereas there is no intrinsic relationship between consecutive measurements at different times. Hence, the space-axis is recommended as the primary operational axis.

### 4.1. Geometric Threshold Method (GTM)

#### 4.1.1. Original Concept

**Figure 3:**
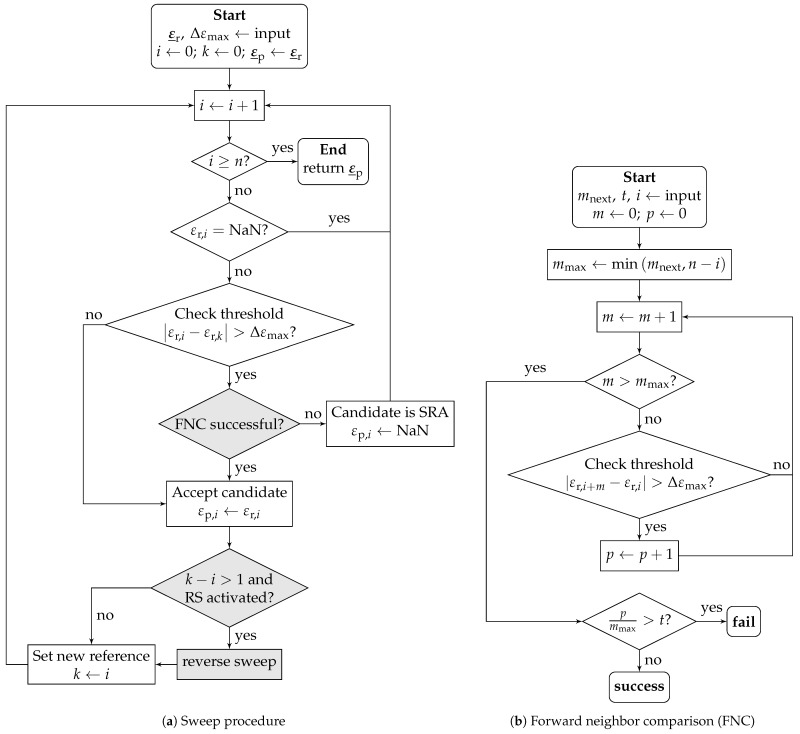
GTM for one-dimensional case, as presented in [19]; additions are highlighted in grey.

The geometric threshold method (GTM) is an algorithm intended to detect and neutralize SRAs based on the comparison of local strain increments [19]. A flowchart representation of the GTM is shown in Figure 3a; additions to the original concept are highlighted in gray. The original (raw) strain array is denoted as ${\underline{\varepsilon }}_{r}$, and the processed array is denoted ${\underline{\varepsilon }}_{p}$. The GTM traverses the strain array with *n* elements and compares the candidate element with index *i* to the current reference element with index *k*. The initial reference element is the first element of the array. Elements that are dropouts are skipped. The candidate is accepted as the new reference element if the absolute strain increment $\Delta \varepsilon =\left|\varepsilon_{i}-\varepsilon_{k}\right|$ from the current candidate element to the reference element does not exceed the threshold $\Delta \varepsilon_{\max}$. Else, the candidate element is considered an SRA and set to NaN in the processed strain array. The modifications of a forward neighbor comparison (FNC) and reverse sweep (RS) are described in Section 4.1.3.

#### 4.1.2. Advantages and Disadvantages

The GTM has the advantages of (i) being a simple algorithm, with a linear time complexity class O(n) and (ii) featuring a threshold with a physical meaning. Hence, the GTM is fast and easy to understand, and its results can be manually verified.

A disadvantage of the original GTM version is that it struggles to recover from large, but legitimate increments in steep strain gradients (which are encountered, for example, in the slopes of crack-induced strain peaks). Since the reference element is updated only when the candidate’s strain value is again within $\varepsilon_{k}\pm \Delta \varepsilon_{\max}$, in strain data with large signal amplitudes, this condition might be fulfilled again only after a considerable distance or not at all. As a result, extensive parts of the processed data might be incorrectly marked as SRAs and replaced with NaN.

#### 4.1.3. Modifications

To overcome the aforementioned flaw, two modifications are introduced. The first modification—reverse sweep (RS)—is a backtracking mode, which is triggered if the previous reference element and the new reference element are not direct neighbors after accepting a candidate as a new reference element. In this case, the “normal” forward sweep is paused, and the strain array is traversed in the reverse direction, starting from the new reference element towards the old reference element. When the old reference point is reached, the forward sweep is resumed.

The original algorithm uses backward-directed comparisons (to previously encountered elements) only. The second improvement—forward neighbor comparison (FNC)—is an additional verification step. A flowchart representation of the FNC is shown in Figure 3b. The FNC is triggered when a candidate is initially flagged as an SRA and compares the strain increment to the elements ahead of the candidate element with the index *i*. In order to handle multiple adjacent SRAs, a configurable number of neighbors $m_{\mathrm{next}}$ are taken into account. Here, *p* is the number of neighbors exceeding the strain increment to the candidate: $\left|\varepsilon_{r,i+m}-\varepsilon_{r,i}\right|>\Delta \varepsilon_{\max}\;\mathrm{for}\;m\in [1,m_{\max}]$. The FNC is successful and the candidate is reaccepted if the ratio $\frac{p}{m_{\max}}$ does not exceed the configurable tolerance ratio *t*. It should be noted that the FNC makes RS mostly dispensable because elements to be reaccepted by RS would already be accepted by FNC.

### 4.2. Outlier-Specific Correction Procedure (OSCP)

#### 4.2.1. Concept

**Figure 4:**
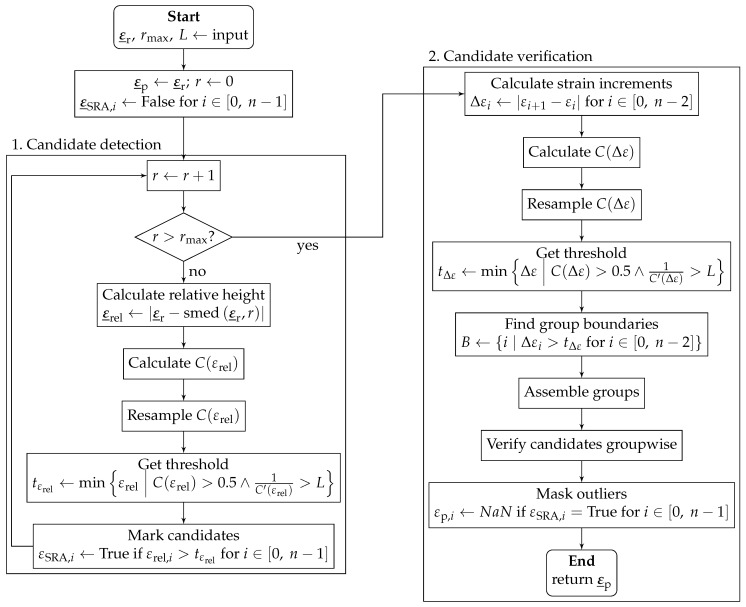
OSCP, adapted from [34].

The outlier-specific correction procedure (OSCP) is an algorithm for outlier identification on regular rasterized topological data [34,35] and can be used within the scope of DSS to detect and neutralize SRAs. The algorithm is structured into two phases: (i) candidate detection and (ii) candidate verification. A flowchart representation of the OSCP is shown in Figure 4.

In the first stage, outlier candidates are detected based on the the elements’ relative height $\varepsilon_{\mathrm{rel}}=\left|\varepsilon_{r}-\mathrm{smed}\left(\varepsilon_{r},r\right)\right|$, which is defined as the absolute difference between the original value $\varepsilon_{r}$ and the value of the central sliding median with its window’s inradius *r*. After the array of relative heights, ${\underline{\varepsilon }}_{\mathrm{rel}}$ is calculated in a vectorized manner, and the cumulative density function $C(\varepsilon_{\mathrm{rel}})$ is established. For reasons of numerical stability, $C(\varepsilon_{\mathrm{rel}})$ is resampled, e.g., using equidistantly spaced quantiles of 1%. The deduction of the threshold $t_{\varepsilon_{\mathrm{rel}}}$ is based on two assumptions: (i) Outliers have a large relative height compared to normal data points. Hence, the threshold has to be located in the upper half ($C\left(\varepsilon_{\mathrm{rel}}\right)>0.5$). (ii) SRAs are few in comparison to normal data points with large distances in $\varepsilon_{\mathrm{rel}}$. Hence, the threshold has to be located in the asymptotic part of $C\left(\varepsilon_{\mathrm{rel}}\right)$, i.e., where the curve is “flat enough”. The second condition is represented by $\frac{1}{C^{\prime }\left(\varepsilon_{\mathrm{rel}}\right)}>L$, with the flatness given as the reciprocal of the cumulative density function’s first derivative $C^{\prime }\left(\varepsilon_{\mathrm{rel}}\right)$ and the flatness level *L* configuration parameter.

An element is flagged as an outlier candidate in the array of boolean values $\varepsilon_{\mathrm{SRA}}$ if its relative height exceeds the threshold: $\varepsilon_{\mathrm{rel},i}>t_{\varepsilon_{\mathrm{rel}}}$. The steps described above (calculation of relative height, determination of the threshold, and candidate flagging) are carried out for variable inradii $r\in \{1,\ldots ,r_{\max}\}$ to detect different-sized outlier clusters. The inradius of the largest window $r_{\max}$ is a configuration parameter that determines the largest detectable outlier cluster. An element enters the verification stage as a candidate if it was flagged in at least one pass among all inradii.

The second stage—outlier verification—is based on the absolute strain increment between neighboring elements Δε. The estimation of the strain increment threshold $t_{\Delta \varepsilon }$ with the cumulated density function C(Δε) is analogous to the first stage. Then, the strain array is divided into groups of contiguous neighboring elements. The set of group boundaries *B* contains the indices of the groups’ last elements, found where the strain increment Δε is larger than the strain increment threshold $\Delta \varepsilon >t_{\Delta \varepsilon }$. While the process of grouping is straightforward for 1D, it is more intricate for 2D and not described in [34,35]. Grouping in 2D is carried out as follows. Firstly, the steps from the strain increment calculation through group boundary detection are carried out separately for each direction. In 2D, indices are an ordered pair. Initial primitive groups are built by iterating over the array (still separated for each dimension) row-wise and column-wise. Before adding the current index to the current group, the conditions for the beginning of a new group are evaluated: (i) the current index is contained in the set of group boundaries *B* or (ii) the end of the array in this direction is reached and the iteration resumes with the first element of the next row or column. Then, the primitive groups are merged when they contain at least one common index, until only groups with pairwise distinct indices are left. Note that this results in non-rectangular shaped 2D groups.

Finally, elements belonging to groups consisting of only candidates are confirmed as SRAs and converted into dropouts, replacing their values with NaN. Candidates in mixed groups (consisting of normal elements and of candidates) are considered normal elements.

#### 4.2.2. Advantages and Disadvantages

The advantageous properties of the OSCP are that it (i) can deal with clustered SRAs, (ii) is direction agnostic, and (iii) supports 2D operation, since it does not depend on a common metric for space and time.

Disadvantageous properties of the OSCP are (i) its complexity with relatively high numerical cost (governed by the repeated sliding median, repeated sorting for the cumulated density function, and the group merging necessary in the 2D case), (ii) the unintuitive flatness level parameter *L*, and (iii) possible instabilities in the threshold estimation because the empiric cumulated density function is not a smooth function, even with resampling.

#### 4.2.3. Modifications

The modifications to the original description presented in [34] are as follows. (i) The cumulative density function *C* is calculated without the intermediate histogram, and *C* is resampled afterwards with a configurable number of quantiles. (ii) In the original use case for surface scanning, both directions have the same unit, and the increments in both directions can be put into the same cumulated density function C(Δε). This is not reasonable for time-series of spatially distributed measurements due to the different meaning of time and space. To support direct 2D operation, the group boundaries are determined for each dimension separately. Hence, the steps from the calculation of Δε through the initial group are carried out for each dimension separately. (iii) The threshold can be set directly, which enables us to skip the determination via the cumulative density function.

### 4.3. Outlier Detection Based on the *z*-Score

#### 4.3.1. Concept

**Figure 5:**
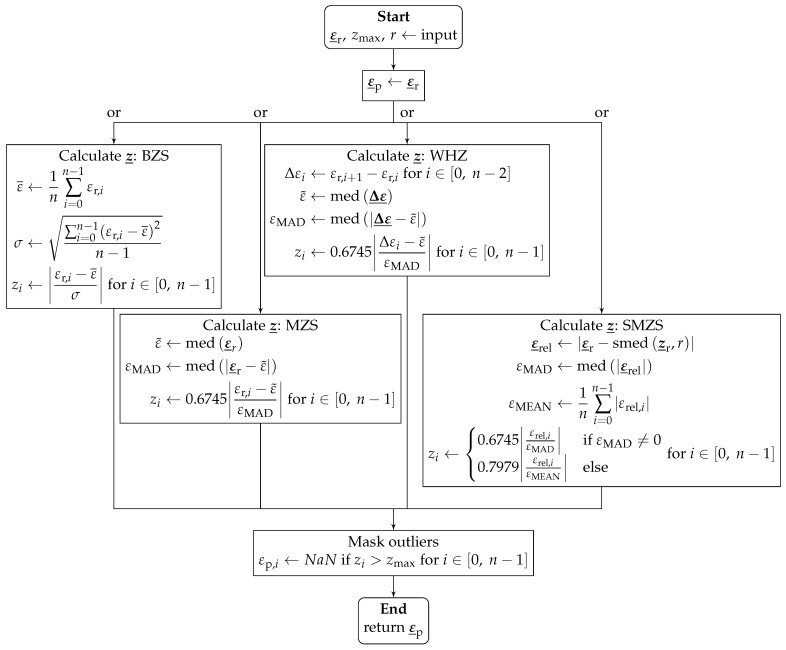
Algorithm of the *z*-score family.

The *z*-score is a statistical measure for the distance of sample values from the sample center. There is a family of simple outlier detection algorithms based on the concept that a value is considered an outlier if its *z*-score exceeds a given threshold $z_{\max}$. Being an outlier measure, the *z*-score can be used to identify and neutralize SRAs. A representation of the general algorithm is shown in Figure 5. There, ${\underline{\varepsilon }}_{r}$ denotes the original (raw) strain array, and ${\underline{\varepsilon }}_{p}$ is the processed strain array.

Four algorithms are presented, which differ in the calculation of the *z*-score. The basic *z*-score (BZS) uses the arithmetic average $\bar{\varepsilon }$ as the sample center and the standard deviation σ as the unit [57].

The modified *z*-score (MZS) uses the median $\tilde{\varepsilon }=\mathrm{med}\left({\underline{\varepsilon }}_{r}\right)$ as the sample center and the median absolute deviation (MAD) $\varepsilon_{\mathrm{MAD}}$ as the unit instead of the standard deviation σ. To obtain results comparable to those of the BZS, the factor 0.6745 is used because the expected value of the MAD is $E_{\mathrm{MAD}}=0.6745\sigma$ for large sets of normally distributed data [57].

The Whitaker–Hayes *z*-score (WHZ) is a modification of the MZS, which uses the array of increments between values $\underline{\Delta \varepsilon }$ instead of the original values [58]. This approach has the benefit that the influences of trends and low-frequency changes in the data are eliminated.

The sliding modified z-score (SMZS) (a modification of the MZS) uses the relative heights ${\underline{\varepsilon }}_{\mathrm{rel}}$ as a basis [21]. The relative heights are given by the difference between the central element $\varepsilon_{r,i}$ and the median of elements in the surrounding window with the inradius *r*. When the mean absolute deviation is used as a fallback option, the correction factor 0.7979 is used instead of 0.6745 because the expected value for the mean absolute deviation is $E_{\mathrm{MEAN}}=0.7979\sigma$ for large sets of normally distributed data [59].

#### 4.3.2. Advantages and Disadvantages

Advantageous properties of the *z*-score-based methods are (i) their algorithmic simplicity and low computational effort with linear time complexity O(n), (ii) that the *z*-score highlights outliers by exaggerating how much an outlier stands out from the normal data, and (iii) that outliers are recognized in relation with the natural variability of the data. Apart from that, the strengths and weaknesses of the algorithm are governed by the strengths and weaknesses of the calculation rule for the *z*-score array.

The BZS has the advantage that it is a common statistical measure. However, a weakness is its vulnerability to the distorting effect that individual outliers have on the *z*-score of all members (caused by the use of the average). Because the BZS takes all members of a sample into account, its use is restricted to stationary data. This makes the BZS unsuitable for non-stationary data, such as DSS data of cracked concrete specimens, where the signal changes over a wide range of values.

The MZS is more robust against outliers due to the use of the median. However, since the MZS uses the complete sample, it is not suitable (like the BZS) to use with DSS data exhibiting large signal variation.

The WHZ solves this problem by operating on the increment. This comes at the expense of false positives, since the *z*-score of normal data adjacent to SRAs is also anomalous. Another weakness is that adjacent SRAs with similar values are not detectable. This might lead to problems in the presence of anomalous areas.

The SMZS intended to solve this problem by taking only the local vicinity into account. The disadvantage is the introduction of an additional parameter *r* for the sliding window, which dependents on the data properties.

### 4.4. Cluster Filter

#### 4.4.1. Concept

**Figure 6:**
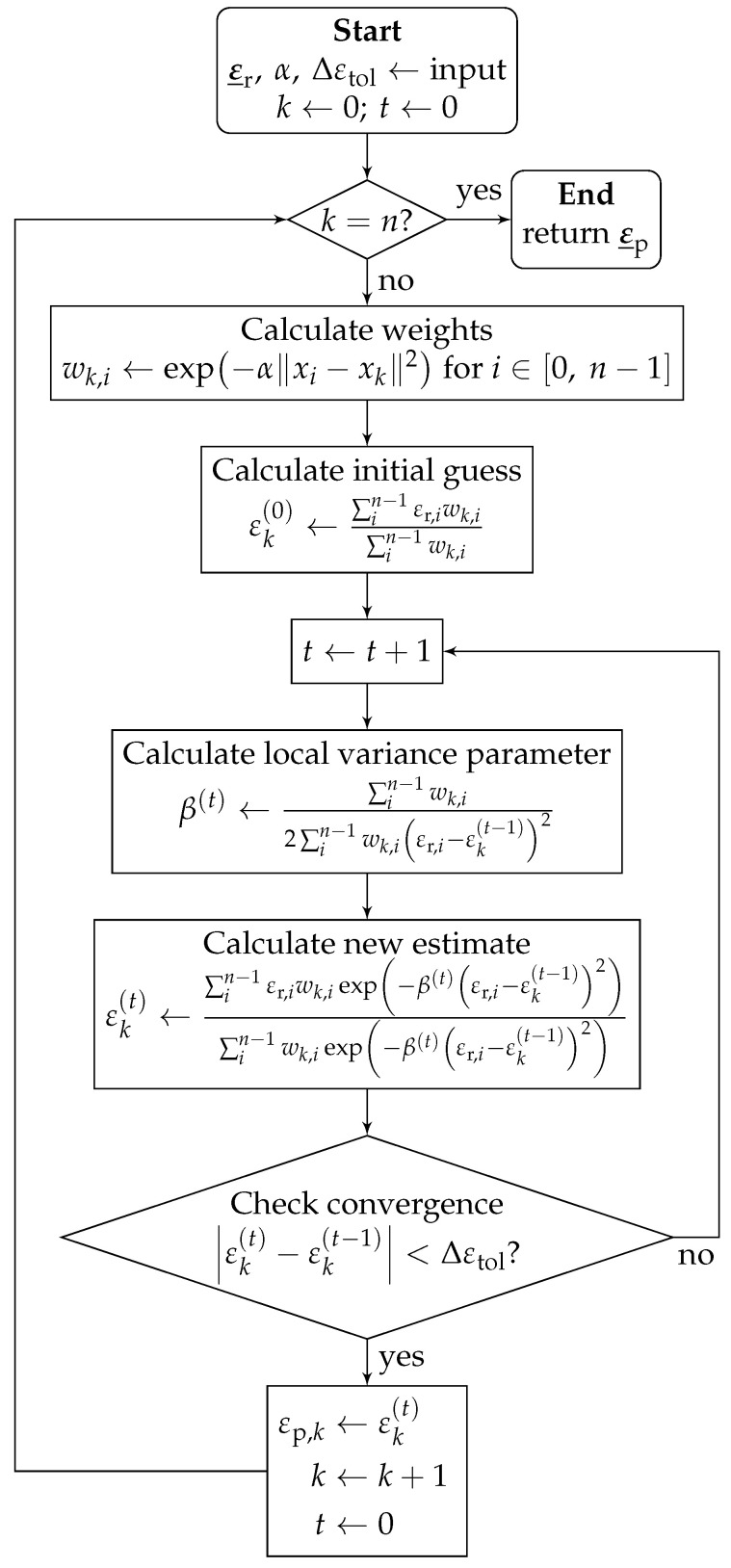
Cluster filter, according to [42].

The cluster filter is an iterative distance-weighted filtering algorithm [42]. It is intended to smooth data by means of outlier resistant noise reduction while preserving local features. A flowchart representation of the cluster filter algorithm is shown in Figure 6. For each element *k* for all *n* elements in the strain array, with its position $x_{k}$ and original strain value $\varepsilon_{r,k}$, the smoothed value is estimated in an iterative process. Here, *n* denotes the total number of elements in the one-dimensional array. The influence weight $w_{k,i}$ of another element identified by the index i∈[0, n−1] on the element *k* falls off exponentially with the squared Euclidean distance ${∥x_{i}-x_{k}∥}^{2}$ multiplied with negative α. The falloff factor α is the main configuration parameter and influences the scale of the cluster filter. The initial estimate is calculated as the weighted average over all elements. In each iteration step *t*, an intermediate parameter $\beta^{\left(t\right)}$ is calculated representing the local variance before proceeding to calculate the next estimate $\varepsilon_{k}^{\left(t\right)}$. The iterative estimation is guaranteed to converge and is stopped if the change in estimation between two consecutive iteration steps falls below the threshold $\Delta \varepsilon_{\mathrm{tol}}$, the second configuration parameter. Then, the value of the final iteration value is stored in the processed strain array ${\underline{\varepsilon }}_{p}$, and the algorithm moves on to the next element.

#### 4.4.2. Advantages, Disadvantages, and Modifications

An advantageous property of the cluster filter is its robustness against SRAs, as their influence is demoted with the parameter β. However, the cluster filter’s high computational cost with a quadratic time complexity class $O(n^{2})$ is a disadvantage.

The algorithm was modified to replace the weight and strain values of dropout elements with 0. This enables the filter to yield decent interpolation for singular or very short dropout fields.

## 5. Benchmarks and Quality Measures

The following benchmarks are used to assess the performance of the presented algorithms to remove c-OFDR measurement principle-related disturbances from DSS data. This section presents the fundamentals of those benchmarks—firstly, the data in Section 5.1, and secondly, the accuracy measures in Section 5.2.

### 5.1. Benchmark Data

Because all real data contain disturbances (related to the measurement principle and physical effects), the ground truth of real measurements cannot be known. In such a case, pre-processing algorithms could only be compared against a reference algorithm—which would naturally perform best in this benchmark (when compared to itself). The benchmarks need to be carried out in a controlled and repeatable environment for a fair performance comparison. Hence, an artificial data set is synthesized, which enables us to compare the result of the pre-processing algorithms to the “true” signal. The “true” signal—hereafter called clean signal (described in Section 5.1.1)—simulates different monitoring use cases. The simulated disturbances with realistic properties—described in Section 5.1.2—enable the transfer of the results to real monitoring data sets.

The disturbed strain array $\underline{\varepsilon_{r}}$—simulating the raw DSS data—is composed as the element-wise superposition of the following components: (i) clean signal ${\underline{\varepsilon }}_{c}$, (ii) noise ${\underline{\varepsilon }}_{n}$, (iii) SRAs ${\underline{\varepsilon }}_{\mathrm{SRA}}$, and (iv) dropouts ${\underline{\varepsilon }}_{d}$:

(1) $${\underline{\varepsilon }}_{r}={\underline{\varepsilon }}_{c}+{\underline{\varepsilon }}_{n}+{\underline{\varepsilon }}_{\mathrm{SRA}}+{\underline{\varepsilon }}_{d}.$$

**Figure 7:**
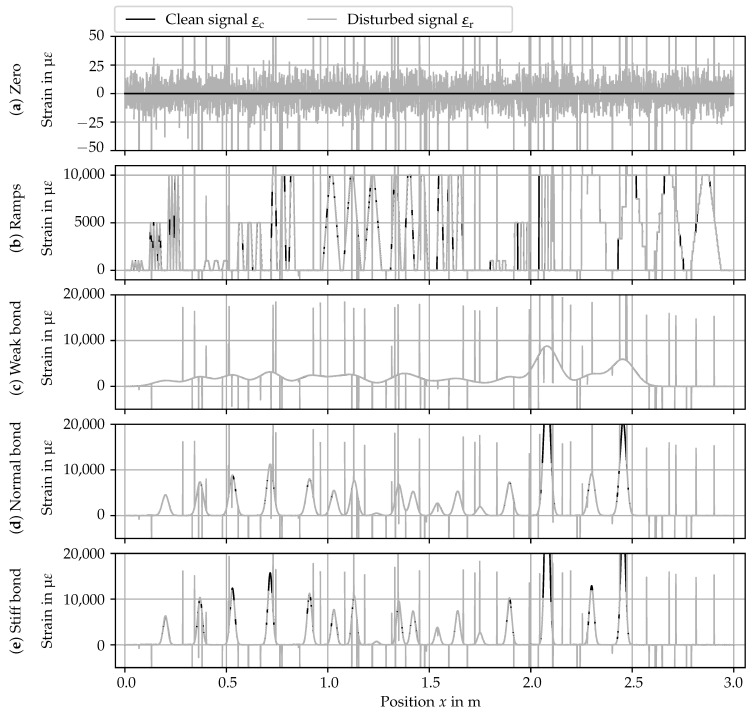
Benchmark data scenarios: (**a**) zero signal; (**b**) ramps; (**c**) strain profile with weak DFOS bond; (**d**) strain profile with medium DFOS bond; (**e**) strain profile with stiff DFOS bond. The data set is available in [60].

The composition of the clean signal ${\underline{\varepsilon }}_{c}$ is described in Section 5.1.1, and the synthesis of the disturbances is presented in Section 5.1.2. A plot of the benchmark data is shown in Figure 7, and the data set was made available as open data [60]. Note the different scaling of the vertical axes.

#### 5.1.1. Clean Signal

To simulate different use cases commonly encountered with DFOS-based DSS monitoring, five different scenarios are generated: (a) constant zero signal, (b) ramps with abrupt changes in the level of the signal, (c) crack pattern with weak bond between the concrete matrix and the DFOS, (d) crack pattern with normal bond between the concrete matrix and the DFOS, and (e) crack pattern with stiff bond between the concrete matrix and the DFOS.

The first scenario (see Figure 7a) simulates a measurement with a stationary signal, encountered in both the space and time domains. In the space domain, such a flat strain profile is encountered in an uncracked concrete specimen (after taring). In the time domain, constant load results in such a stationary time-series for one gage. Hence, the clean signal is the zero vector.

The second scenario (see Figure 7b) simulates ramps and steps encountered with, e.g., varying cross-sectional geometries in steel construction. The spikes and jumps are deliberately modeled to exaggerate potentially difficult cases for the algorithms. Hence, it is expected that this scenario is difficult for the algorithms, resulting in a high number of misidentifications.

The last three scenarios (see Figure 7c–e) simulate strain curves obtained in the use case of the DFOS-based crack monitoring of concrete structures. The crack positions and widths are fixed. From Figure 7c–e, the bond stiffness between the DFOS’ core and the substrate increases, resulting in increasingly pronounced strain peaks. The bond stiffness can be described by means of the DFOS sensitivity [61]:

(2) $$\gamma =\frac{\varepsilon_{\max}}{w_{\mathrm{cr}}}$$

with the crack width $w_{\mathrm{cr}}$ and the maximum strain reading $\varepsilon_{\max}$. The higher the sensitivity, the stiffer the bond between optical fiber and substrate [61]. Values for this benchmark are chosen based on the results of preliminary studies [15,36]. A weak bond is simulated with $\gamma =7\;m^{-1}$, which is associated with a layered robust DFOS. A medium bond is simulated with $\gamma =25\;m^{-1}$, corresponding to a monolithic robust DFOS. A stiff bond is simulated with $\gamma =35\;m^{-1}$, corresponding to a filigree DFOS with an acrylate coating.

The clean signal ${\underline{\varepsilon }}_{c}$ for the strain profiles (Figure 7c–e) is the superposition of several peak shape functions. Due to its resemblance to strain peaks in real DSS data, a Gaußian function is chosen as the base peak shape function

(3) $$\phi \left(x,\mu ,\sigma \right)=\frac{1}{\sigma \sqrt{2\pi }}\exp\left(-\frac{{\left(x-\mu \right)}^{2}}{2\sigma^{2}}\right)$$

with the expected value μ and standard deviation σ. Considering Equation (2), σ becomes

(4) $$\sigma =\frac{1}{\gamma \sqrt{2\pi }}.$$

By inserting Equation (4) into Equation (3), replacing the expected value μ with the position of the crack $x_{cr}$, and scaling with the crack width $w_{\mathrm{cr}}$, the strain function for the *k*th crack $\varepsilon_{k}$ becomes

(5) $$\varepsilon_{k}\left(x,w_{\mathrm{cr},k},\gamma ,x_{\mathrm{cr},k}\right)=w_{\mathrm{cr},k}\gamma \times \exp\left(-\frac{{\left(x-x_{\mathrm{cr},k}\right)}^{2}}{2{\left(\frac{1}{\gamma \sqrt{2\pi }}\right)}^{2}}\right).$$

Finally, the clean signal is synthesized by superposing all strain peaks for all *k* cracks:

(6) $${\underline{\varepsilon }}_{c}\left(\underline{x}\right)=\sum_{k}{\underline{\varepsilon }}_{k}\left(\underline{x},w_{k},\gamma ,x_{\mathrm{cr},k}\right).$$

Equation (6) is applied in a vectorized manner for the positional array $\underline{x}$, which is generated in the range of 0 m to 3 m with a spatial resolution (gage pitch) of 0.65 mm. The parameters $x_{\mathrm{cr},k}$, $w_{\mathrm{cr},k}$, and $\varepsilon_{\max,k}$ for different γ values are given in Table 1.

#### 5.1.2. Disturbance Parameters

The parameters for the disturbances—noise, SRAs, and dropouts—are chosen to simulate measurement data of mediocre quality. The noise component is simulated with a standard normal distribution with the expected value μ=0 με and a standard deviation σ=10 με drawn as a consecutive sequence with a random number generator.

SRAs are simulated using a two-step process. Firstly, the positions of the SRAs are randomly distributed through a Bernoulli trial using a 5% probability for an element to be an SRA. Then, the SRA values are sampled randomly from a data set extracted from real experiments [60,62] and added to the signal.

An element is a dropout if any of the following criteria is fulfilled. (i) The element is chosen in a Bernoulli trial with a dropout rate of 10%. (ii) The absolute value exceeds $|{\underline{\varepsilon }}_{c}|>12,000\;\mu \varepsilon$, simulating the ODiSI’s absolute measurement range [22]. (iii) The absolute strain increment exceeds $|\Delta \varepsilon_{c}|>975\;\mu \varepsilon$, simulating the ODiSI’s technical limit for the measurable the strain gradient. This strain increment threshold is related to the ODiSI’s maximum measurable strain gradient of 1480 με/mm for a gage pitch of 0.65 mm [36]. Values of dropout elements are replaced with NaN in the strain signal. Note that the dropout takes precedence if a reading is both a dropout and a SRA.

Noise, randomly distributed dropouts (due to the Bernoulli process criterion) and SRAs are fixed across all scenarios for comparability, i.e., the values are generated once and added to all scenarios. However, the dropouts simulating the technical limits of the ODiSI differ between the scenarios.

### 5.2. Performance Measures

#### 5.2.1. SRA Detection Accuracy Measures

The SRA detection accuracy is estimated based on the rate of false positives (normal data classified as SRAs) and false negatives (SRAs classified as normal data) in relation to the correct number of SRAs. Because the confusion matrix does not take the severity of the misidentification into account, another measure is developed: the weighted confusion measure (WCM). The WCM is a penalty function that reflects both frequency and the impact of misidentifications. Hence, the WCM is higher as more misidentifications occur and with larger identification mistakes. The WCM has two components: one for false positives ($\alpha_{\mathrm{WCM}}$) and one for false negatives ($\beta_{\mathrm{WCM}}$).

Regarding false negatives, the larger the missed SRA, the larger is severity of the mistake. The penalty for a false negative at the index *i* is $\varepsilon_{\mathrm{SRA},i}$ the SRA-value added to the clean signal, which is known for the synthetic benchmark data. Then, $\beta_{\mathrm{WCM}}$ is the sum over all penalties:

(7) $$\beta_{\mathrm{WCM}}=\sum_{i}\left\{\begin{array}{cc} \varepsilon_{\mathrm{SRA},i} & \mathrm{if}\;\mathrm{false}-\mathrm{negative},\\ 0 & \mathrm{else}. \end{array}\right.$$

For false positives, the opposite is the case: the false positiveis the worse, the more similar an element is to its surrounding. However, since $\varepsilon_{\mathrm{SRA},i}=0$ false positives, an additional penalty value $p_{\mathrm{WCM}}$ is introduced. It is compared to the actual absolute average strain increments $\Delta \varepsilon_{l,i}=\varepsilon_{i}-\varepsilon_{i-1}$ and $\Delta \varepsilon_{r,i}=\varepsilon_{i+1}-\varepsilon_{i}$ to the left and right neighbors of the noisy strain signal’s element. The penalty for a false positive is the absolute difference between $p_{\mathrm{WCM}}$ and the average of those two strain increments. Then, $\alpha_{\mathrm{WCM}}$ is the sum over all penalties:

(8) $$\alpha_{\mathrm{WCM}}=\sum_{i}\left\{\begin{array}{cc} p_{\mathrm{WCM}}-\left|\Delta \varepsilon_{l,i}-\Delta \varepsilon_{r,i}\right| & \mathrm{if}\;\mathrm{false}-\mathrm{positive},\\ 0 & \mathrm{else}. \end{array}\right.$$

To avoid artifacts, $p_{\mathrm{WCM}}$ should be set about the largest expected false positive SRAs. In the following benchmarks, $p_{\mathrm{WCM}}=10,000\;\mu \varepsilon$ is used.

#### 5.2.2. Filter Quality Measures

The filtering accuracy is assessed based on the deviation between the clean signal ${\underline{\varepsilon }}_{c}$ and the processed signal ${\underline{\varepsilon }}_{p}$ with the positional index *i* and the total number of gages *n*. Then, the mean error (ME) is

(9) $${\Delta \varepsilon }_{\mathrm{ME}}=\frac{1}{n}\sum_{i}^{n}\left|\varepsilon_{c,i}-\varepsilon_{p,i}\right|.$$

Another measure—which emphasizes larger derivations—is the root mean square error (RMSE):

(10) $${\Delta \varepsilon }_{\mathrm{RMSE}}=\sqrt{\frac{1}{n}\sum_{i}^{n}{\left(\varepsilon_{c,i}-\varepsilon_{p,i}\right)}^{2}}.$$

#### 5.2.3. Crack Width Error Measure

The accuracy of dropout reconstruction is assessed with the relative crack width estimation error

(11) $$\Delta w_{\mathrm{cr},\mathrm{rel}}=\frac{w_{\mathrm{cr},\mathrm{num}}-w_{\mathrm{cr},\mathrm{ref}}}{w_{\mathrm{cr},\mathrm{ref}}}$$

with $w_{\mathrm{cr},\mathrm{num}}$ being the crack width integrated from the pre-processed (reconstructed) strain peak and $w_{\mathrm{cr},\mathrm{ref}}$ being the reference crack width.

## 6. Results and Discussion

This section presents the carried out benchmarks and is divided into four subsections. The three first subsections are dedicated to SRA detection algorithms (Section 6.1), noise reduction (Section 6.2), and the reconstruction of strain peaks with dropouts (Section 6.3). In each of those subsections, the specific benchmark setup is described followed by a presentation and discussion of the results. General limitations of the benchmark are pointed out in Section 6.4. All benchmarks were carried out using fosanalysis.

### 6.1. SRA Detection Accuracy

In this study, the SRA detection algorithms were applied on the benchmark data and their performances were evaluated.

#### 6.1.1. Setup

**Figure 8:**
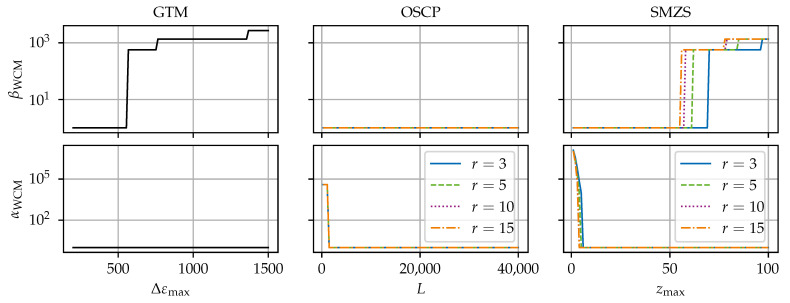
SRA detection accuracy for the algorithms for scenario (a) zero.

**Figure 9:**
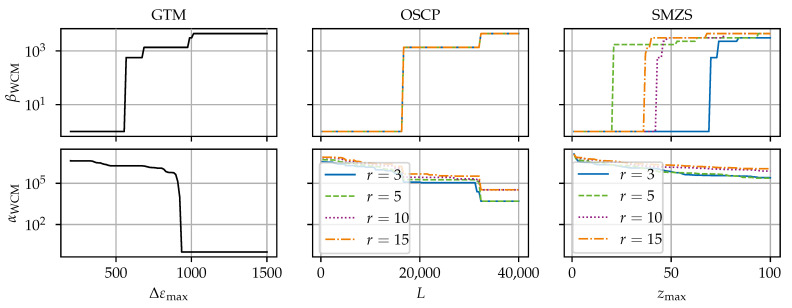
SRA detection accuracy for the algorithms for scenario (b) ramps.

**Figure 10:**
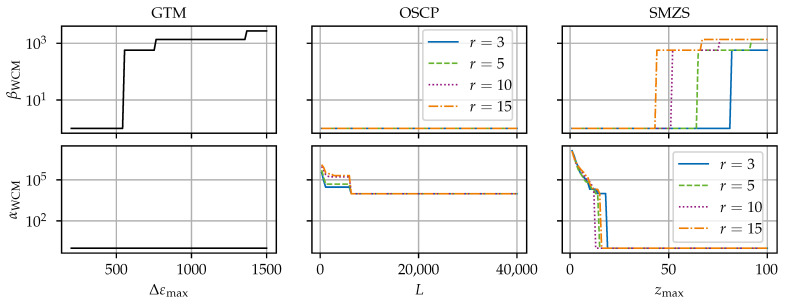
SRA detection accuracy for the algorithms for scenario (c) weak bond.

**Figure 11:**
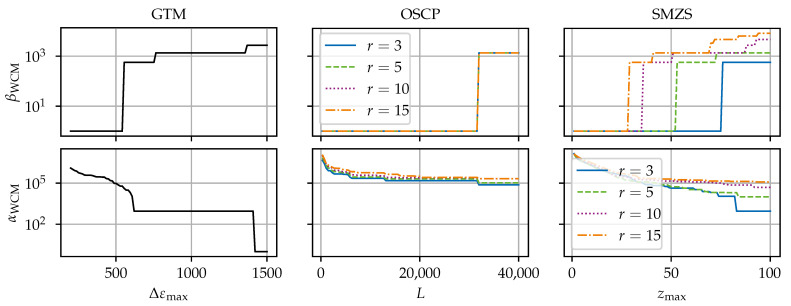
SRA detection accuracy for the algorithms for scenario (d) normal bond.

**Figure 12:**
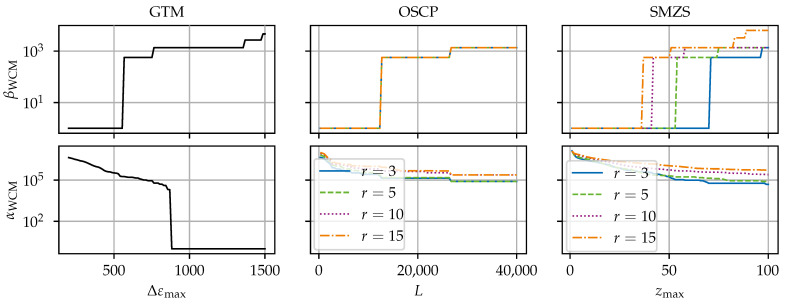
SRA detection accuracy for the algorithms for scenario (e) stiff bond.

Figure 8, Figure 9, Figure 10, Figure 11 and Figure 12 compare the algorithms GTM, OSCP, and SMZS in terms of their accuracy for the five benchmark scenarios examined, as shown in Figure 8, Figure 9, Figure 10, Figure 11 and Figure 12. The *x*-axes show the threshold value (according to the respective definition). The threshold is varied in the intervals (i) $\Delta \varepsilon_{\max}\in [300,\;1500]$ for the GTM, (ii) L∈[0, 40,000] for the OSCP, and (iii) $z_{\max}\in [1.0,\;100]$ for the SMZS. For the GTM, FNC with a range of $m_{\mathrm{next}}=1$ and a tolerance t=0 and RS were activated. The OSCP and SMZS additionally depend on the radius of the underlying sliding windows, which is varied r∈{3, 5, 10, 15}. The legend is used for both the OSCP and SMZS. The *y*-axes show the WCM components for false negative $\beta_{\mathrm{WCM}}$ and false positive $\alpha_{\mathrm{WCM}}$ misidentifications depending on the configuration, with lower values for $\alpha_{\mathrm{WCM}}$ and $\beta_{\mathrm{WCM}}$ indicating a better accuracy. The logarithmic *y*-axes allow for a direct comparison. To circumvent the problem that 0 cannot be displayed in a logarithmic scale, 1 is added to all values as the base line.

#### 6.1.2. Scenario Comparison

The optimal algorithm configuration is dependent on the data set [21]. Hence, the different scenarios are used to identify the strengths and weaknesses of the algorithms. The trade-off between the rate of false negatives to false positives is seen in the general tendency that higher thresholds result in smaller $\alpha_{\mathrm{WCM}}$ but larger $\beta_{\mathrm{WCM}}$.

The results in scenarios (a) and (c) (see Figure 8 and Figure 10) are very similar. Both scenarios (a) and (c) have a low variance in the signal, so the SRAs stand out clearly, making them the easiest scenarios for the SRA detection. All algorithms perform well, and there exists a configuration range for perfect results with neither false positives nor false negatives for the GTM and SMZS but not for the OSCP in scenario c, where the OSCP reports more false positives.

The results for the both crack scenarios (d) and (e) (see Figure 11 and Figure 12) are closely related. With a higher variance in both scenarios (d) and (e), no configuration can be found for neither false negatives nor false positives. Still, scenario (e) is more difficult due to the dropouts and steeper gradients, resulting in a slightly tighter configuration leeway. In the plot for the GTM, the value used to simulate the technical strain gradient limit is clearly visible represented in the drop in $\alpha_{\mathrm{WCM}}$.

Scenario (b) (see Figure 9) stands out as the most difficult one among the five scenarios, and the algorithms have a considerably higher $\alpha_{\mathrm{WCM}}$ and $\beta_{\mathrm{WCM}}$. There is no configuration yielding perfect or near-perfect results. The cause for these difficulties are the steep ramps and sharp tips of the triangular ramps. Here, the random dropouts in the ramps impact the OSCP and SMZS. The large variance in the ramps reduces the *z*-score of the SRAs in the SMZS. Avoiding enormous amounts of false positives in the ramps with the GTM requires $\Delta \varepsilon_{\max}$ to be larger that any genuine strain increment. Hence, scenario (b) would require an infeasible high threshold, preventing the GTM from identifying smaller SRAs.

#### 6.1.3. Algorithm Comparison

With a larger radius for the OSCP and SMZS, fewer false negatives but more false positives are generated. However, the sensitivity to the radius seems to be higher for the number of false positives than the number of false negatives. The explanation for this is that the benchmark data contain HL-SRAs only and no anomalous areas. Tips of peaks are more likely flagged as SRAs the larger the radius is.

The presence of dropouts in the vicinity of SRAs generally increases the probability of misidentifications and makes configuration more difficult. Hence, the largest influence due to dropout is encountered in scenarios (d) and (e). The classification of the OSCP and SMZS is unreliable for the *r* gages neighboring wide continuous dropout fields because the reduced number of values makes the sliding median less robust. Hence, locally extreme values (e.g., in the flanks) become false positives, and SRAs embedded in the dropout field cannot be detected and become false negatives.

##### GTM

Compared to the other SRA detection algorithms, the GTM has the advantages (i) that it has only one primary configuration parameter ($\Delta \varepsilon_{\max}$), which has a physical meaning (intuitive to understand) and a comfortable configuration corridor; (ii) that it retains regular data at the edge of compact dropout fields; (iii) that it should be able to detect even HF-SRAs with appropriate settings; and (iv) its low computational requirements. However, it struggles with steep inclinations (seen in scenario b). The lower limit for the threshold is the technical limit of the ODiSI, up to which strain increments should be considered as regular. The upper limit is given by the lowest SRAs contained in the data set. Hence, the threshold should be set to about $\Delta \varepsilon_{\max}=1000\;\mu \varepsilon$, which proved reliable for all scenarios.

##### OSCP

For the most scenarios, the OSCP takes the middle ground between the GTM and SMZS, but has slightly better results than the other two algorithms in scenario (b). Compared to the other two algorithms, the OSCP’s advantages are (i) the data-driven adaptive threshold with rich configuration options, which is not as intuitive to understand; (ii) that it can identify clustered SRAs; and (iii) it being native in 2D operation mode, which could find more HF-SRAs but at the cost of a serious performance impact.

However, the OSCP has weaknesses in that it tends to flag normal data in the vicinity (up to $r_{\max}$) of compact dropout fields in peak flanks, cf. scenarios (d) and (e). This behavior is due to the fact that the median is distorted and the values close to the dropout field are the most extreme ones in the sliding window. These additional false positives contribute to the growths of the dropout field. As rule of thumb, the larger the $r_{\max}$, the longer the band of false positives, but the larger the threshold and the fewer false positives. False negatives are most likely when enclosed by large compact dropout fields.

Another drawback is the high computational cost of the OSCP because the sliding median is carried out multiple times over the entire data set. This will become more relevant the higher that $r_{\max}$ is.

To reduce $\beta_{\mathrm{WCM}}$, the radius should be larger than $r_{\max}>2$, but since $\beta_{\mathrm{WCM}}$ increases with the radius, *r* should not be excessively large. Hence, a value of $r_{\max}=5$ is appropriate for the benchmark data. The flatness should be set at about L∈[15,000, 40,000]. The best-performing setup for the benchmark data at hand would be $r_{\max}=5$ and L=20,000.

Instead of the data-driven estimation of *t*, the OSCP allows the user to set *t* directly as well. In this case, the OSCP yields an accuracy comparable to that of the GTM, and $t=\Delta \varepsilon_{\max}=1000\;\mu \varepsilon$ seems to be a good threshold, but the disadvantageous canceling of genuine values at dropout fields remains.

##### SMZ

As visible in Figure 8, Figure 9, Figure 10, Figure 11 and Figure 12, with the exception of Figure 10, the results of the SMZS and OSCP are similar. The SMZS’s configuration range is tighter than that of the OSCP in most scenarios. While the SMZS perform worse than the OSCP in scenario (b), the SMZS performs better than the OSCP in crack pattern scenarios.

The *z*-score algorithm family comprises several variants [21]. Each variant implements options for different data set characteristics. Advantages of the *z*-score algorithms are (i) that the threshold $z_{\max}$ is based on the statistical distribution of the data; (ii) that a dynamic threshold ($z_{\max}$ (that varies over the data set) for signals with varying characteristics is possible [21]; (iii) that it can take the complete measurement into account, which works best for nearly constant signals; and (iv) that it supports native 2D operation.

On the other hand, the SMZS comes with some disadvantages as well: (i) its tendency to flag false positives at the edges of dropout fields in the peak slopes (up to $\frac{r}{2}$), (ii) its configuration corridor for good results is tighter than that of GTM with more sensitive parameters (especially the radius), and (iii) its limited capability in anomalous areas.

The numerical cost of the SMZS is between those of the GTM and OSCP. The best-performing parameter combination for the benchmark data would be r=5 and $z_{\max}=40$.

### 6.2. Noise Reduction Benchmark

#### 6.2.1. Setup

This benchmark is used to compare (i) the sliding average, also known as sliding mean, (ignoring NaNs); (ii) the sliding median (ignoring NaNs); and (iii) the cluster filter for their effectiveness in reducing noise and their suitability in neutralizing SRAs. Two cases are investigated. The first case considers noise as the only disturbance type: the “dirty” signal is composed of the clean signal ${\underline{\varepsilon }}_{c}$ and the noise component ${\underline{\varepsilon }}_{c}$. The second case additionally incorporates SRAs and dropouts and uses the benchmark data as shown in Figure 7 to test the robustness of these three methods. This benchmark will highlight the importance of dedicated SRA detection algorithms and show that the sliding average is particularly inappropriate for removing SRAs.

The algorithm’s filter width parameter (which configures how far one element influences the filtered signal) was varied to investigate the sensitivity. The filter width parameter for the sliding filters is the window radius, which was varied between r∈[0, 10]. The higher the *r*, the higher the width. The filter width parameter for the cluster filter is the scaling factor α, which was varied $\alpha \in [10^{3},\;10^{6}]$. The lower the α, the higher the width. Additionally, the cluster filter’s tolerance convergence criterion is fixed at 0.1, and the filling of the dropouts is deactivated. Note that a radius of r=0 or α=∞ is equivalent to no filtering.

#### 6.2.2. Results

**Figure 13:**
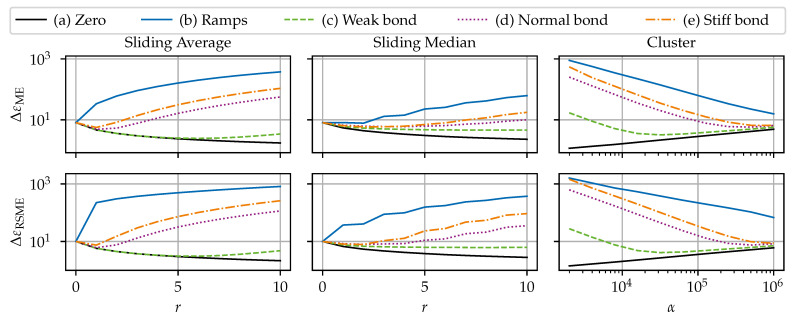
Results of filter benchmarks for case 1: noisy signal without SRAs or dropouts.

**Figure 14:**
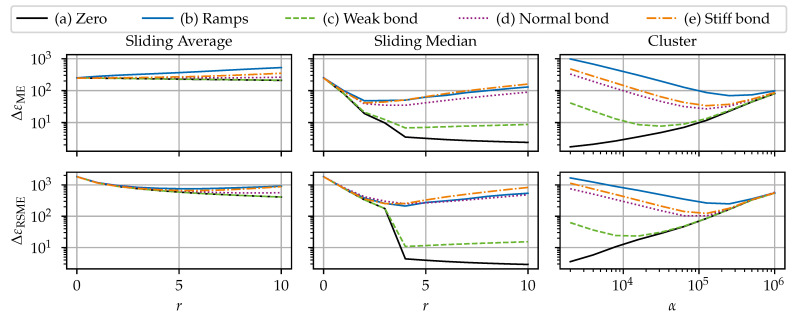
Results of filter benchmarks for case 2: noisy signal with SRAs and dropouts.

The accuracy of the filtering methods is assessed by means of the ${\Delta \varepsilon }_{\mathrm{ME}}$ according to Equation (9), and the ${\Delta \varepsilon }_{\mathrm{RMSE}}$, according to Equation (10). The combined interpretation of both ${\Delta \varepsilon }_{\mathrm{ME}}$ and ${\Delta \varepsilon }_{\mathrm{RMSE}}$ enables a more detailed assessment. For reference, the noisy signal has the error measures ${\Delta \varepsilon }_{\mathrm{ME}}=8\;\mu \varepsilon$ and ${\Delta \varepsilon }_{\mathrm{RMSE}}=10\;\mu \varepsilon$. Note the logarithmic scale for the error measures (*y*-axis) and the parameter α (*x*-axis) for the cluster filter in Figure 13 and Figure 14.

##### Case 1: Noise Only

The first benchmark case’s results are shown in Figure 13. Overall, all three filters show similar results. It is notable that both ${\Delta \varepsilon }_{\mathrm{ME}}$ and ${\Delta \varepsilon }_{\mathrm{RMSE}}$ show the same qualitative behavior, indicating that there is no redistribution of errors. The filter width-dependent behavior results can be distinguished into three families: (i) the quasi-stationary (i.e., no or very little variance in the clean signal) scenario a), where the errors approach 0 with increasing filter width; (ii) the dynamic (i.e., mild to strong variance in the clean signal) scenarios (c), (d), and (e), where errors first decrease up to a certain filter width, but after that increase again; and (iii) the sharp-edged (i.e., strong variance in the clean signal with sharp edges) scenario (b), where the error is strictly increasing with an increasing filter width. The sliding average performs slightly better than the sliding median in scenario (a), as the sliding average is statistically optimal filter with white (i.e., normally distributed random) noise with respect to reaction sharpness [47].

For the medium dynamic scenarios, the error measures first decrease, but increase again with increasing range. The points of the minimum errors depend on the sharpness of the changes in the signal. Eventually, the errors exceed the raw signals’ errors at the point where the negative effect of signal manipulation dominates the smoothing effect. This shows the tight limits for the beneficial use of filtering. The beneficial range of the sliding median is larger than the sliding averages’ one.

**Figure 15:**
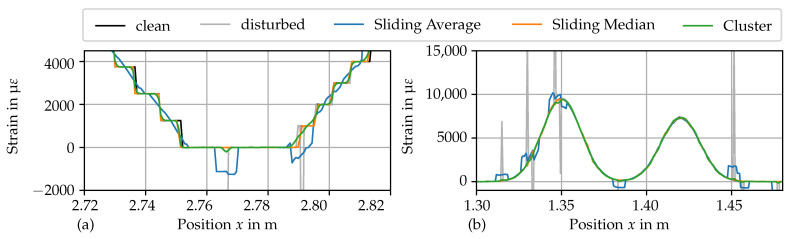
Section of strain data pre-processed with the filters: sliding average (r=5), sliding median (r=5), cluster filter ($\alpha =1.3\times 10^{5}$). (**a**) Scenario (b), (**b**) scenario (d).

The results in scenario (b) show that the sliding median preserves edges better than the sliding average. The sliding average and the cluster filter round out curves, resulting in a distorted signal; see Figure 15.

##### Case 2: Noise, SRAs, and Dropouts

The second benchmark case’s results are shown in Figure 14. Due to the simulated dropouts, the different scenarios have varying values for the ${\Delta \varepsilon }_{\mathrm{ME}}$ of the unfiltered result from 245.8 με to 252.4 με and for the ${\Delta \varepsilon }_{\mathrm{RMSE}}$ from 1814.8 με to 1836.3 με.

The results of the algorithms differ notably for the second case (with dropouts and SRAs). Firstly, for the sliding average, it is visible that the ${\Delta \varepsilon }_{\mathrm{ME}}$ is on a relatively constant, high level. The non-stationary scenarios do not have a beneficial range for the filter width. The ${\Delta \varepsilon }_{\mathrm{ME}}$ increase comes from the “interpolation” effect on boundaries of large compact dropout fields, which grows in strength with the filter width. Conversely, the sliding average has a smooth interpolation effect on isolated dropouts. However, the ${\Delta \varepsilon }_{\mathrm{RMSE}}$ is reduced with increasing filter width. This shows that the sliding average does not eliminate the errors introduced by the SRA. Instead, the sliding average redistributes the error value of the SRAs to the adjacent *r* elements. Hence, an SRA is softened and results in bumps in the signal; see Figure 15. This has a serious implication, as those bumps might be indistinguishable from legitimate local effects (e.g., cracks) and alter the actual signal.

A considerable reduction in both ${\Delta \varepsilon }_{\mathrm{ME}}$ and ${\Delta \varepsilon }_{\mathrm{RMSE}}$ is achieved when employing the sliding median. The drastic drop in errors indicates that the sliding median remediates the errors introduced by the SRAs. The sliding median’s filtered signal is not affected by isolated SRA. Again, the points of minimum errors depend on the variance in the clean signal and the error measure, with the corresponding radius varying r∈[2, 5]. The increasing part is again due to the distortion governed by the distortion on the edges of dropout fields; see Figure 15.

The cluster filter takes the middle ground between the sliding average and the sliding median. Similar to to the sliding average, the cluster filter softens SRAs to bumps in the resulting signal. However, due to the large dissimilarity of SRAs with the surrounding data, the cluster filter assigns a smaller weight to SRAs. Hence, the disruptive influence of SRAs is drastically reduced. Similarly to the other algorithms, the cluster filter exhibits a boundary effect on large dropouts fields. In comparison to the other filters, the cluster filter requires enormous numerical effort.

##### Filtering Example

Figure 15 shows examples of raw and filtered data for a visual comparison. Figure 15a shows a section from scenario (b), highlighting the different capabilities of the filters to preserve edges. Figure 15b shows a section from scenario (d), where the distorting effect on the edge of the dropout field is visible. It is visible in both figures how the sliding average and the cluster filter smooth out SRAs and the sliding median cancels out SRAs.

##### Recommendations

As with anomaly detection, the best parameters are use case-dependent. The sliding average is a very solid choice for noisy, but SRA free data, but are inappropriate for eliminating SRA. While less prone to SRAs, the same judgment holds for the cluster filter. In the case of data with SRAs, it is recommended to employ a dedicated SRA detection algorithm (or alternatively, the sliding median) instead. The SRA detection algorithm could be followed by an optional filter pass. However, the filtering pass is not always necessary. Filtering might improve the signal quality but bears the risk of signal distortion if overdone. As an aside, this investigation showed that there was no benefit to chaining a sliding median and a sliding average.

### 6.3. Dropout Reconstruction

#### 6.3.1. Setup

This section is dedicated to comparing two simple, yet common repair methods: (i) linear interpolation and (ii) spline interpolation using an Akima spline. For demonstration purposes, a Gaußian curve, normalized to $x_{\mathrm{cr}}=0$, $\varepsilon_{\max}=1$, and σ=1, was chosen. The influence of scaling and sensitivity is negligible for the sake of this benchmark.

In the first case of the benchmark, the steepest parts of the slope—located around the inflections points—are erased. Dropouts in the peak flanks caused by strain gradients that are too steep are the most common cause of dropouts [21,36]. With the factor *t* for the widths of the dropout field in terms of the curve’s standard deviation σ computed according to Equation (4), all elements $\varepsilon_{i}$ within tσ around the curve’s inflection points $|x_{i}\pm \sigma |\;<\;t\sigma$ are set to NaN.

**Figure 16:**
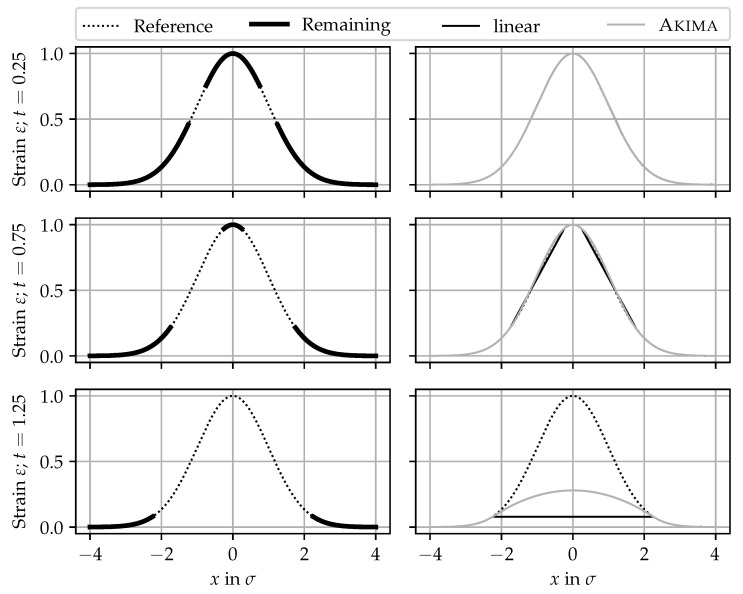
Left column: normalized strain peak with applied dropouts; right column: reconstructed strain peak with (i) t=0.25, (ii) t=0.75, and (iii) t=1.25. The right-hand-side plot details the highlighted part in the left-hand-side plot.

In the second case of the benchmark, the highest part of the peak is erased by setting all elements $\varepsilon_{i}$ to NaN, for which $|x_{i}|\;<\;\left(1+t\right)\sigma$ holds. In other words, the part of the tip that would remain in the first case when t<1 is erased as well; see Figure 16. Then, the dropouts are replaced with interpolated values, and the crack widths are calculated by integrating the strain profile [31]. Finally, the accuracy of the reconstruction is evaluated using Equation (11).

#### 6.3.2. Results

Figure 16 shows some strain profiles for selected t∈{0.25, 0.75, 1.25} for the first case. Both cases are identical for t>1. The left-hand side shows the reference signal and the signal with the dropouts. The right-hand-side plots show the same strain profiles interpolated using the two approaches. While the difference is not visible or very small for t<1, the difference between the reference and the reconstructed signal in the third example, where the peak tip is missing, is striking.

**Figure 17:**
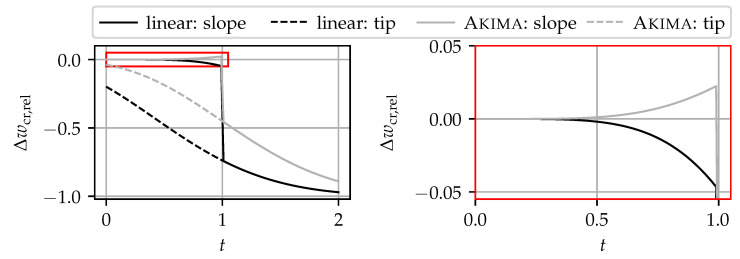
Error in crack widths $\Delta w_{\mathrm{cr},\mathrm{rel}}$ for linear and Akima spline interpolation, depending on the amount of dropout *t*. The right-hand-side plot is the highlighted detail of the left plot.

In Figure 17, the relative crack width error according to Equation (11) is plotted against *t*. Solid lines correspond to the first case (erased slopes), and dashed lines correspond to the second benchmark case (erased tip). As already mentioned, both benchmark cases are the same for t>1.0. However, for t<1.0, they differ fundamentally.

In the second benchmark case, the crack width is underestimated by both approaches, and the errors increase continuously with increasing *t*. As expected, the Akima spline is closer to the reference than the linear interpolation. Note that for the second benchmark case, the peak tip between the inflection points is already erased for t=0.0.

In the first benchmark case, the errors stay close to 0 until the critical point t=1.0. Then, the complete peak tip is lost, and both approaches lead to prone errors. For better visibility, the error of the first benchmark case’s errors for t∈[0.0, 1.0]—where the peak tip is still present—is magnified in the right-hand-side plot. It is visible that the errors do not exceed ±5%. These observations are consistent with those from experimental tests [36]. While the linear interpolations tend to underestimate the crack width, the Akima spline tends to overestimate it, which is on the safe side. Whether the crack width is underestimated or overestimated depends on the general shape function of the crack. For an exponential peak function [63], both approaches might overestimate the actual crack width. In general, the Akima spline offers a smaller deviation from the reference curve than the linear interpolation.

The conclusions from this benchmark are as follows. As long as the tip of a strain peak is preserved, the reconstruction results are acceptable with both interpolation methods. Although the Akima spline has an overall better fit than the linear interpolation, both approaches are not able to reconstruct the complete upper peak tip with reasonable error. For reconstructing strain peaks with a severe amount of dropout, more sophisticated estimation approaches are required. Those approaches—based on mechanical models [63,64], fitting analytical functions [36], or machine learning models [65,66]—could leverage the knowledge of DFOS sensitivity for specific combinations of sensor, adhesive, and installation methods.

At the moment, preserving the strain peak tips is imperative for reliable crack monitoring. This conclusion imposes implications for both the “hardware” and the “software” part. Regarding the “hardware”, it is most important to choose a sensor-adhesive combination appropriate for the measuring task, e.g., based on preliminary crack pattern simulation [36]. For the “software”, it is most important to not remove the peak tips in the pre-processing, especially in the SRA masking stage. In particular, when only a few gages with valid values are left, distinguishing them from SRAs is challenging.

### 6.4. Limitations

Some limitations of this study shall be pointed out. The benchmark contained several scenarios to simulate different use cases. However, real data might contain further interfering influences caused by physical effects, such as inhomogeneities in the concrete material [43], effects of local bonds [16,33,44], or temperature changes [67]. Because accounting for those physical effects (e.g., by compensation measures) is another step in the data evaluation; these were not taken into account in the benchmark. The SRAs and dropouts were distributed independently of each other in the benchmark data. However, it was observed that SRAs and dropouts do have a dependence and that it is more likely that an SRA neighbors a dropout or another SRA than a genuine value [62]. Additionally, the simulation did not take HF-SRAs into account. Only a rather simple approach was taken to simulate the dropouts due to technical limitations, as a more detailed investigation can be found in [36]. The algorithms may therefore perform differently on real data. The benchmark was carried out on only one instance of the benchmark data, only on one-dimensional data. However, the algorithms could utilize additional information when operating in a native 2D mode.

## 7. Conclusions

Even with the best monitoring setup, DSS measurement data might contain serious signal disturbances. Depending on their severity, these disturbances make further data processing and information extraction difficult or even impossible. Therefore, the pre-processing of the raw data with advanced approaches is required. The three different disturbance types found in DSS data—SRAs, dropouts, and noise—were investigated in this study. Their causes and commonly applied remediation approaches were discussed. Selected pre-processing approaches were discussed in detail, implemented and benchmarked with a synthetic data set. A generic pre-processing workflow for DSS data was proposed and implemented into the free open source software framework fosanalysis. The following conclusions could be drawn.

### 7.1. SRA Detection

In general, it is recommended to use a dedicated SRA detection algorithm. All three investigated algorithms (GTM, OSCP, and SMZS) can detect SRAs with acceptable reliability. Only a few minor and hard-to-detect SRAs were undetected. Given the benchmark results, the GTM would be equally or slightly preferred over SMZS, with the OSCP taking third place. The algorithms differ mostly in their tendency to false positives in the vicinity of dropout fields and their numerical costs. For all algorithms, a trade-off between false negatives and false positives was observed, and suitable parameter ranges were identified. However, false positives are not problematic, unless they remove primary features (e.g., the peak tips) of the signal. Hence, some manual quality control is still recommended.

### 7.2. Filtering

Filtering has a limited beneficial range with an optimum in the trade-off between noise removal and preserving local effects. However, some use cases might not require filtering at all. For example, the noise of 10 με is unproblematic for crack width monitoring [16]. All three investigated filters (sliding average, sliding median, and the cluster filter) work well on SRA free signals. In this case, the benchmark results of the sliding median are comparable to those of the sliding average. In the second case, where strain curves with SRAs and dropouts were analyzed, the sliding median was shown to be stable against solitary anomalies with more extreme deviations from the true signal. As these outliers are the main challenge for the subsequent processing steps, the sliding median is often the better choice over the cluster filter or the sliding average.

### 7.3. Reconstruction

Dropouts most commonly occur in the flanks of strain peaks due to exceeding the technical limitations of the ODiSI. The reconstruction of dropout sections using simple interpolation methods (such as linear and Akima spline interpolation) yields good results as long as the strain peaks tips are preserved. In this case, the relative error for crack width calculation stays within 5%. However, if the strain peak tip is lost, the associated crack width is drastically underestimated. Hence, the best strategy is to avoid excessive absolute strain values and strain gradients by selecting an appropriate DFOS-adhesive combination according to the monitoring task [36]. Other approaches could yield more accurate peak reconstruction results, e.g., based on machine learning [65,68], mechanical models [63,69], or numerical simulations [70,71].

### 7.4. Final Remarks

Since the requirements for the pre-processing workflow are specific to the data set at hand, there is no “one size fits all” solution for pre-processing. Hence, a flexible workflow is proposed with these recommended steps (although the order might require changes):

- Use a dedicated SRA detection algorithm;
- Use downsampling along the time axis with the median as an aggregation function to leverage its noise reduction feature which is robust against HL-SRAs when analyzing static loading situations;
- Use the sliding median as a smoothing filter;
- Use an Akima spline for interpolation.

Pre-processing cannot replace high-quality raw data in the first place. Maintaining the best possible quality of raw data is a critical task in the design phase of DFOS-based monitoring systems. For example, preserving the strain peak tips is critical for crack monitoring. In the value chain of data processing, the trustworthiness of the final result is limited to the lowest trustworthiness of all the links in the entire chain. Hence, no matter what pre-processing is used, if the raw data are of poor quality, the results cannot be trusted.

## Figures and Tables

**Table 1:** Crack pattern parameters for the simulated strain profiles Figure 7c–e.

Number	Position	Crack Width	Maximum Strain $\varepsilon_{\max,k}$ in με		
k	$x_{\mathrm{cr},k}$ **in** m	$w_{\mathrm{cr},k}$ **in** μm	$\gamma =7\;m^{-1}$	$\gamma =25\;m^{-1}$	$\gamma =35\;m^{-1}$
1	0.2	181	1267	4525	6335
2	0.37	295	2065	7375	10,325
3	0.53	354	2478	8850	12,390
4	0.716	450	3150	11,250	15,750
5	0.91	321	2247	8025	11,235
6	1.03	220	1540	5500	7700
7	1.13	308	2156	7700	10,780
8	1.24	21	147	525	735
9	1.35	273	1911	6825	9555
10	1.42	211	1477	5275	7385
11	1.54	109	763	2725	3815
12	1.64	213	1491	5325	7455
13	1.75	77	539	1925	2695
14	1.895	293	2051	7325	10,255
15	2.08	1253	8771	31,325	43,855
16	2.3	369	2583	9225	12,915
17	2.455	836	5852	20,900	29,260

## Data Availability

The investigated algorithms were implemented into the Python software framework fosanalysis, which is made available as free open source software at https://github.com/TUD-IMB/fosanalysis (accessed on 18 November 2024). The benchmark data along with the Python scripts for generating the data and carrying out the benchmarks were made available as open data [60]. The experimental data on the characteristics of SRAs were made available as open data [62].

## Abbreviations

BZS	basic *z*-score
c-OFDR	coherent optical frequency domain reflectometry
DFOS	distributed fiber optic sensor
DSS	distributed strain sensing
FNC	forward neighbor comparison
GTM	geometric threshold method
HF-SRA	harmful strain reading anomaly
HL-SRA	harmless strain reading anomaly
MAD	median absolute deviation
MZS	modified *z*-score
OSCP	outlier-specific correction procedure
NaN	not a number
ODiSI	optical distributed sensor interrogator
RS	reverse sweep
SHM	structural health monitoring
RMSE	root mean square error
ME	mean error
SRA	strain reading anomaly
SMZS	sliding modified *z*-score
SSQ	spectral shift quality
WCM	weighted confusion measure
WHZ	Whitaker–Hayes *z*-score
$\alpha_{\mathrm{WCM}}$	weighted confusion measure for false positives
$\beta_{\mathrm{WCM}}$	weighted confusion measure for false negatives

## References

[1] Cheng L., Cigada A., Zappa E., Gilbert M., Lang Z.Q. (2024). Dynamic monitoring of a masonry arch rail bridge using a distributed fiber optic sensing system. J. Civ. Struct. Health Monit..

[2] Bednarski Ł., Sieńko R., Howiacki T., Zuziak K. (2022). The Smart Nervous System for Cracked Concrete Structures: Theory, Design, Research, and Field Proof of Monolithic DFOS-Based Sensors. Sensors.

[3] Howiacki T., Sieńko R., Bednarski Ł., Zuziak K. (2023). Structural monitoring of concrete, steel, and composite bridges in Poland with distributed fibre optic sensors. Struct. Infrastruct. Eng..

[4] Vorwagner A., Kwapisz M., Lienhart W., Winkler M., Monsberger C., Prammer D. (2021). Verteilte Rissbreitenmessung im Betonbau mittels faseroptischer Sensorik—Neue Anwendung von verteilten faseroptischen Messsystemen. Beton-und Stahlbetonbau.

[5] Monsberger C., Bauer P., Buchmayer F., Lienhart W. (2022). Large-scale distributed fiber optic sensing network for short and long-term integrity monitoring of tunnel linings. J. Civ. Struct. Health Monit..

[6] Zhang X., Zhu H., Jiang X., Broere W. (2024). Distributed fiber optic sensors for tunnel monitoring: A state-of-the-art review. J. Rock Mech. Geotech. Eng..

[7] Brezzi L., Schenato L., Cola S., Fabbian N., Chemello P., Simonini P. (2023). Smart Monitoring by Fiber-Optic Sensors of Strain and Temperature of a Concrete Double Arch Dam. Geotechnical Engineering in the Digital and Technological Innovation Era.

[8] Wang D.Y., Zhu H.H., Wu B., Ye X., Wang J., Tan D.Y., Shi B. (2024). Performance evaluation of underground pipelines subjected to landslide thrust with fiber optic strain sensing nerves. Acta Geotech..

[9] Lienhart W., Monsberger C.M., Buchmayer F. (2022). How to Make a Self-sensing House with Distributed Fiber Optic Sensing. European Workshop on Structural Health Monitoring.

[10] Wijaya H., Rajeev P., Gad E. (2021). Distributed optical fibre sensor for infrastructure monitoring: Field applications. Opt. Fiber Technol..

[11] Kishida K., Imai M., Kawabata J., Guzik A. (2022). Distributed Optical Fiber Sensors for Monitoring of Civil Engineering Structures. Sensors.

[12] Wang J., Garg A., Satyam N., Zhussupbekov A., Sushkova S. (2024). DFOS Technology in Geoengineering Monitoring in the Past 35 Years: A Bibliometric Analysis. Sensors.

[13] Barrias A., Casas J., Villalba S. (2016). A Review of Distributed Optical Fiber Sensors for Civil Engineering Applications. Sensors.

[14] Bado M.F., Casas J.R. (2021). A Review of Recent Distributed Optical Fiber Sensors Applications for Civil Engineering Structural Health Monitoring. Sensors.

[15] Herbers M., Richter B., Gebauer D., Claßen M., Marx S. (2023). Crack Monitoring on Concrete Structures—Comparison of Various Distributed Fiber Optic Sensors with Digital Image Correlation Method. Struct. Concr..

[16] Richter B., Herbers M., Marx S. (2023). Crack monitoring on concrete structures with distributed fiber optic sensors—Toward automated data evaluation and assessment. Struct. Concr..

[17] Paul A., Sanio D., Mark P. (2024). Monitoring tendon breaks in concrete structures at different depths using distributed fiber optical sensors. e-J. Nondestruct. Test..

[18] Howiacki T., Sieńko R., Bednarski Ł., Zuziak K. (2023). Crack Shape Coefficient: Comparison between Different DFOS Tools Embedded for Crack Monitoring in Concrete. Sensors.

[19] Bado M.F., Casas J.R., Gómez J. (2021). Post-processing algorithms for distributed optical fiber sensing in structural health monitoring applications. Struct. Health Monit..

[20] Janiak T., Becks H., Camps B., Classen M., Hegger J. (2023). Evaluation of distributed fibre optic sensors in structural concrete. Mater. Struct..

[21] Ulbrich L., Stührenberg J., Al-Zuriqat T., Chillón Geck C. (2024). Detection and elimination of strain reading anomalies in distributed strain sensing readings. Proceedings of the Tagungsband des 35. Forum Bauinformatik 2024.

[22] Luna Innovations Inc. (2020). User’s Guide ODiSI 6: Optical Distributed Sensor Interrogator Model ODiSI 6: User’s Guide ODiSI 6 Software. https://lunainc.com/sites/default/files/assets/files/resource-library/ODiSI%206100%20User%20Guide.pdf.

[23] Alj I., Quiertant M., Khadour A., Grando Q., Benzarti K. (2021). Environmental Durability of an Optical Fiber Cable Intended for Distributed Strain Measurements in Concrete Structures. Sensors.

[24] Bremer K., Alwis L.S.M., Zheng Y., Weigand F., Kuhne M., Helbig R., Roth B. (2019). Durability of Functionalized Carbon Structures with Optical Fiber Sensors in a Highly Alkaline Concrete Environment. Appl. Sci..

[25] Lemcherreq Y., Galkovski T., Mata-Falcón J., Kaufmann W. (2022). Application of Distributed Fibre Optical Sensing in Reinforced Concrete Elements Subjected to Monotonic and Cyclic Loading. Sensors.

[26] Gómez J., Casas J.R., Villalba S. (2020). Structural Health Monitoring with Distributed Optical Fiber Sensors of tunnel lining affected by nearby construction activity. Autom. Constr..

[27] Bado M.F., Casas J.R., Barrias A. (2018). Performance of Rayleigh-Based Distributed Optical Fiber Sensors Bonded to Reinforcing Bars in Bending. Sensors.

[28] Luna Innovations Inc. (2017). User’s Guide ODiSI-B: Optical Distributed Sensor Interrogator Model ODiSI-B: User’s Guide ODiSI-B Software 5.2.0. https://www.advancedphotonix.com/wp-content/uploads/2014/05/ODiSI-B-Users-Guide.pdf.

[29] Barrias A., Casas J.R., Villalba S. (2018). Embedded Distributed Optical Fiber Sensors in Reinforced Concrete Structures-A Case Study. Sensors.

[30] Chamoin L., Farahbakhsh S., Poncelet M. (2022). An educational review on distributed optic fiber sensing based on Rayleigh backscattering for damage tracking and structural health monitoring. Meas. Sci. Technol..

[31] Fischer O., Thoma S., Crepaz S. (2019). Distributed fiber optic sensing for crack detection in concrete structures. Civ. Eng. Des..

[32] Li Y., Sharif-Khodaei Z. (2023). Accuracy of Distributed Strain Sensing with Single-Mode Fibre in Composite Laminates under Thermal and Vibration Loads. Struct. Control. Health Monit..

[33] Galkovski T., Lemcherreq Y., Mata-Falcón J., Kaufmann W. (2021). Fundamental Studies on the Use of Distributed Fibre Optical Sensing on Concrete and Reinforcing Bars. Sensors.

[34] Bin Ismail M.F., Yanagi K., Fujii A. (2010). An outlier correction procedure and its application to areal surface data measured by optical instruments. Meas. Sci. Technol..

[35] Ismail M.F.B., Jaafar T.R., Che Mat S., Pahmi M.A.A.H. (2014). Evaluation of Outlier Specific Correction Procedure for Areal Surface Texture. Appl. Mech. Mater..

[36] Herbers M., Richter B., Marx S. (2024). Rayleigh-based crack monitoring with distributed fiber optic sensors—Experimental study on the interaction of spatial resolution and sensor type. Struct. Control Health Monit..

[37] Ansari F., Libo Y. (1998). Mechanics of Bond and Interface Shear Transfer in Optical Fiber Sensors. J. Eng. Mech..

[38] Her S.C., Huang C.Y. (2011). Effect of Coating on the Strain Transfer of Optical Fiber Sensors. Sensors.

[39] Weisbrich M., Holschemacher K., Bier T. (2020). Comparison of different fiber coatings for distributed strain measurement in cementitious matrices. J. Sens. Sens. Syst..

[40] Akima H. (1970). A New Method of Interpolation and Smooth Curve Fitting Based on Local Procedures. J. ACM.

[41] Weisbrich M., Holschemacher K., Bier T. (2021). Validierung verteilter faseroptischer Sensorik zur Dehnungsmessung im Betonbau. Beton-und Stahlbetonbau.

[42] Lou S., Tang D., Zeng W., Zhang T., Gao F., Muhamedsalih H., Jiang X., Scott P.J. (2020). Application of Clustering Filter for Noise and Outlier Suppression in Optical Measurement of Structured Surfaces. IEEE Trans. Instrum. Meas..

[43] Weisbrich M., Messerer D., Holschemacher K. (2023). The Challenges and Advantages of Distributed Fiber Optic Strain Monitoring in and on the Cementitious Matrix of Concrete Beams. Sensors.

[44] Koschemann M., Curbach M., Marx S., Hofmann J., Plizzari G. (2022). Investigation of local bond behavior using distributedoptical fiber sensing. Proceedings of the Bond in Concrete—Bond, Anchorage, Detailing: 5th International Conference.

[45] Wheeler L.N., Pannese E., Hoult N.A., Take W.A., Le H. (2018). Measurement of distributed dynamic rail strains using a Rayleigh backscatter based fiber optic sensor: Lab and field evaluation. Transp. Geotech..

[46] Xiang G., Sun A., Liu Y., Gao L. (2024). An improved denoising method for *Φ*-OTDR signal based on the combination of temporal local GMCC and ICEEMDAN-WT. Opt. Fiber Technol..

[47] O’Haver T.C. (2023). A Pragmatic Introduction to Signal Processing 2023.

[48] Smith S.W. (2003). Digital Signal Processing: A Practical Guide for Engineers and Scientists.

[49] Ershov I.A., Stukach O.V., Trubin I.V., Gladyshev S.A. Features of Digital Filters in Raman DTS. Proceedings of the 2023 Dynamics of Systems, Mechanisms and Machines (Dynamics).

[50] Piątek B., Howiacki T., Kulpa M., Siwowski T., Sieńko R., Bednarski Ł. (2023). Strain, crack, stress and shape diagnostics of new and existing post-tensioned structures through distributed fibre optic sensors. Measurement.

[51] Monsberger C.M., Lienhart W. (2021). Distributed Fiber Optic Shape Sensing of Concrete Structures. Sensors.

[52] Ouellet S.M., Dettmer J., Lato M.J., Cole S., Hutchinson D.J., Karrenbach M., Dashwood B., Chambers J.E., Crickmore R. (2024). Previously hidden landslide processes revealed using distributed acoustic sensing with nanostrain-rate sensitivity. Nat. Commun..

[53] Liu H., Zhang S., Coulibaly A.A.S., Cheng J., DeJong M.J. (2021). Monitoring Reinforced Concrete Cracking Behavior under Uniaxial Tension Using Distributed Fiber-Optic Sensing Technology. J. Struct. Eng..

[54] Ma K.F., von Specht S., Kuo L.W., Huang H.H., Lin C.R., Lin C.J., Ku C.S., Wu E.S., Wang C.Y., Chang W.Y. (2024). Broad-band strain amplification in an asymmetric fault zone observed from borehole optical fiber and core. Commun. Earth Environ..

[55] Ershov I.A., Stukach O.V. Choice of Wavelet for Filtering of Signal from Fiber-Optic Temperature Sensor. Proceedings of the 2022 Moscow Workshop on Electronic and Networking Technologies (MWENT).

[56] Ershov I.A., Stukach O.V., Myasnikova N.V. Features of the Implementation of the Extremal Filtration Method in the Distributed Optic-Fiber Temperature Sensor. Proceedings of the 2021 International Seminar on Electron Devices Design and Production (SED).

[57] Iglewicz B., Hoaglin D.C. (1993). How to Detect and Handle Outliers.

[58] Whitaker D.A., Hayes K. (2018). A simple algorithm for despiking Raman spectra. Chemom. Intell. Lab. Syst..

[59] Pham-Gia T., Hung T. (2001). The mean and median absolute deviations. Math. Comput. Model..

[60] Richter B. (2024). Artificial data set for benchmarking pre-processing algorithms for distributed fiber optic strain data.

[61] Zhang S., Liu H., Coulibaly A.A.S., DeJong M. (2021). Fiber optic sensing of concrete cracking and rebar deformation using several types of cable. Struct. Control. Health Monit..

[62] Ulbrich L. (2024). Statistical Evaluation of Strain Reading Anomalies in Distributed Fiber Optic Strain Sensing Measurements.

[63] Bassil A., Chapeleau X., Leduc D., Abraham O. (2020). Concrete Crack Monitoring Using a Novel Strain Transfer Model for Distributed Fiber Optics Sensors. Sensors.

[64] Hong C., Rao W., Qiu T., Chen X., Dai J., Wu C., Li M., Chen W. (2024). Monitoring and assessment of mechanical response of large-scale prefabricated structures in underground subway stations during construction process. Measurement.

[65] Liu Y., Bao Y. (2023). Automatic interpretation of strain distributions measured from distributed fiber optic sensors for crack monitoring. Measurement.

[66] Liu Y., Bao Y. (2023). Intelligent monitoring of spatially-distributed cracks using distributed fiber optic sensors assisted by deep learning. Measurement.

[67] Murray M.J., Murray J.B., Ogden H.M., Redding B. (2022). Dynamic temperature-strain discrimination using a hybrid distributed fiber sensor based on Brillouin and Rayleigh scattering. Opt. Express.

[68] Venketeswaran A., Lalam N., Wuenschell J., Ohodnicki P.R., Badar M., Chen K.P., Lu P., Duan Y., Chorpening B., Buric M. (2021). Recent Advances in Machine Learning for Fiber Optic Sensor Applications. Adv. Intell. Syst..

[69] Zhang S., Liu H., Cheng J., DeJong M.J. (2020). A mechanical model to interpret distributed fiber optic strain measurement at displacement discontinuities. Struct. Health Monit..

[70] Alj I., Quiertant M., Khadour A., Grando Q., Terrade B., Renaud J.C., Benzarti K. (2020). Experimental and Numerical Investigation on the Strain Response of Distributed Optical Fiber Sensors Bonded to Concrete: Influence of the Adhesive Stiffness on Crack Monitoring Performance. Sensors.

[71] Billon A., Hénault J.M., Quiertant M., Taillade F., Khadour A., Martin R.P., Benzarti K. (2015). Qualification of a distributed optical fiber sensor bonded to the surface of a concrete structure: A methodology to obtain quantitative strain measurements. Smart Mater. Struct..

